# Analysis of Virus and Host Proteomes During Productive HSV-1 and VZV Infection in Human Epithelial Cells

**DOI:** 10.3389/fmicb.2020.01179

**Published:** 2020-05-29

**Authors:** Werner J. D. Ouwendijk, Lennard J. M. Dekker, Henk-Jan van den Ham, Tihana Lenac Rovis, Erik S. Haefner, Stipan Jonjic, Jürgen Haas, Theo M. Luider, Georges M. G. M. Verjans

**Affiliations:** ^1^Department of Viroscience, Erasmus MC, Rotterdam, Netherlands; ^2^Neurology, Erasmus MC, Rotterdam, Netherlands; ^3^Enpicom B.V., ‘s-Hertogenbosch, Netherlands; ^4^Center for Proteomics and Department of Histology and Embryology, Faculty of Medicine, University of Rijeka, Rijeka, Croatia; ^5^Experimental and Translational Oncology, University Medical Center Mainz, Mainz, Germany; ^6^Division of Infection and Pathway Medicine, University of Edinburgh, Edinburgh, United Kingdom

**Keywords:** varicella-zoster virus, herpes simplex virus 1, mass-spectrometry, epidermal growth factor, retinal pigment epithelial cells

## Abstract

Herpes simplex virus 1 (HSV-1) and varicella-zoster virus (VZV) are two closely related human alphaherpesviruses that persistently infect most adults worldwide and cause a variety of clinically important diseases. Herpesviruses are extremely well adapted to their hosts and interact broadly with cellular proteins to regulate virus replication and spread. However, it is incompletely understood how HSV-1 and VZV interact with the host proteome during productive infection. This study determined the temporal changes in virus and host protein expression during productive HSV-1 and VZV infection in the same cell type. Results demonstrated the temporally coordinated expression of HSV-1 and VZV proteins in infected cells. Analysis of the host proteomes showed that both viruses affected extracellular matrix composition, transcription, RNA processing and cell division. Moreover, the prominent role of epidermal growth factor receptor (EGFR) signaling during productive HSV-1 and VZV infection was identified. Stimulation and inhibition of EGFR leads to increased and decreased virus replication, respectively. Collectively, the comparative temporal analysis of viral and host proteomes in productively HSV-1 and VZV-infected cells provides a valuable resource for future studies aimed to identify target(s) for antiviral therapy development.

## Introduction

Most adults worldwide are infected with the human alpha-herpesviruses (αHHV) herpes simplex virus 1 (HSV-1) and varicella-zoster virus (VZV) (Arvin and Gilden, 2013; Roizman et al., 2013). Both viruses are typically acquired during childhood and subsequently establish a lifelong latent infection in sensory neurons. Periodic reactivation and anterograde transport of virus to innervating mucosae leads to productive infection of epithelial cells and dissemination to naïve individuals. HSV-1 is the causative agent of oral and genital herpes, and an important cause of ocular disease and viral encephalitis (Roizman et al., 2013). VZV causes varicella (chickenpox) as a primary infection and herpes zoster (HZ; shingles) upon reactivation from latency, frequently complicated by chronic pain (post-herpetic neuralgia) (Arvin and Gilden, 2013). While VZV vaccines have been developed to prevent varicella in children and HZ in adults (Takahashi et al., 1974; Oxman et al., 2005; Lal et al., 2015), HZ vaccines are not widely used due to costs, partial protection and painful side effects (Oxman, 2010; Cunningham, 2016). No vaccines are currently available to prevent HSV disease. Insight into fundamental biological processes shared by HSV-1 and VZV could provide valuable target(s) to develop novel antiviral therapies.

Herpesvirus gene expression is tightly regulated by viral and host proteins (Pellett and Roizman, 2013). The HSV-1 genome is 152 kb in size and contains over 90 unique genes, encoding more than 84 proteins, that are expressed in a temporally cascade fashion and traditionally classified as immediate-early (α), early (β) and late (γ) transcripts (Roizman et al., 2013). The VZV genome is 125 kb in size and contains at least 71 unique protein-encoding genes 1 (Arvin and Gilden, 2013; Depledge et al., 2018). Transcriptional regulation of VZV genes is ill-defined, mostly due to the highly cell-associated nature of VZV that precludes synchronized infections (Arvin and Gilden, 2013). Analyses of selected viral proteins in newly VZV-infected cells, using cell cultures inoculated with VZV-infected cells, suggest coordinated expression of VZV genes (Reichelt et al., 2009; Lenac Roviš et al., 2013). The temporally regulated expression of HSV-1 and VZV proteins most likely results in a dynamic interaction between viral and host proteomes during productive replication.

RNA-sequencing of HSV-1-infected cells provided essential insights into the complexities of the viral transcriptome and viral (de-)regulation of host transcription (Harkness et al., 2014; Rutkowski et al., 2015; Wyler et al., 2017). Nevertheless, post-transcriptional modifications - and viral regulation thereof (Elliott et al., 2018) - can significantly alter virus and host proteomes. Proteomic approaches have been used to study the function of individual HSV-1 proteins and composition of HSV-1 virus particles (Wagner and Deluca, 2013; Lippe, 2014). However, few studies investigated the global and dynamic changes of viral proteins during productive HSV-1 infection (Antrobus et al., 2009; Berard et al., 2015; Kulej et al., 2017) and, to our knowledge, none were performed for productive VZV infection. Importantly, intra-viral protein networks of herpesviruses are evolutionary conserved and interact broadly with the cellular proteome (Uetz et al., 2006; Fossum et al., 2009). Therefore, identification of host factors that are critical to replication of related herpesviruses could provide targets for development of novel, broadly applicable antiviral therapies. Using recently developed protocols to generate high-titer cell-free VZV (Schmidt-Chanasit et al., 2008; Sloutskin et al., 2013), we aimed to analyze the temporal changes in virus and host protein expression during productive HSV-1 and VZV infection in the same cell line.

## Materials and Methods

### Antibodies

The following antibodies were used for flow cytometry: mouse anti-ICP0 (clone 11066), UL39 (clone 7602), UL29 (clone 23F10) and gC (clone 1001) all kindly provided by the University of Glasgow (United Kingdom), mouse anti-gD (clone AP7) and gH:gL complex (clone LP11) obtained from the University of Cambridge (United Kingdom), mouse anti-PLAA (clone E-1) and mouse-anti-LOX (clone F-8) (both Santa Cruz Biotechnology), allophycocyanin-conjugated goat anti-mouse IgG (BD Biosciences). Antibodies used for WB: mouse anti-VZV ORF4 (clone 4.05), ORF8 (clone 8.04), ORF31 (clone 31c.09, ORF36 (clone 36.14) and ORF63 (clone 63.08) (Lenac Roviš et al., 2013), rabbit polyclonal anti-GFP (A11122) and mouse anti-SPARC (clone ON1-1) (both: Thermo Fisher Scientific), mouse anti-β-actin (clone C4), mouse anti-PLAA (clone E-1), mouse anti-NLRP14 (clone F-8) and mouse anti-LOX (clone H-10) (all from: Santa Cruz Biotechnology), mouse anti-EGFR (clone 13/EGFR; BD Biosciences) and rabbit anti-phospho-EGFR (Tyr1068) (clone D7A5; Cell Signaling Technology), mouse anti-HSV-1 ICP0 (clone 11066), mouse anti-ICP4 (clone 10462) and mouse anti-glycoprotein D (clone AP16) all provided by the University of Glasgow (United Kingdom), rabbit anti-US1 (ICP22) polyclonal antibody R77, mouse anti-UL29 (ICP8) (clone 10A3; Santa Cruz Biotechnology), IRDye 680- and IRDye 800-conjugated goat anti-rabbit and goat anti-mouse antibodies (Westburg). The following antibodies were used for immunofluorescent staining: mouse anti-PLAA (clone E-1), mouse-anti-LOX (clone F-8) and mouse anti-NLRP14 (cone H-10) (all from: Santa Cruz Biotechnology), rabbit polyclonal anti-GFP (A11122) and mouse anti-SPARC (clone ON1-1) (both: Thermo Fisher Scientific), Alexa Fluor 488 (AF488)-conjugated goat anti-rabbit IgG and AF594 goat anti-mouse IgG (both: Thermo Fisher Scientific).

### Cells and Viruses

Non-immortalized human retinal pigment epithelial ARPE-19 cells (American Type Culture collection ID: CRL-2302) were cultured in a 1:1 ratio (vol/vol) of DMEM and Ham’s F12 nutrient mixture (both Invitrogen) supplemented with 10% heat-inactivated fetal bovine serum (FBS; Sigma Aldrich) and antibiotics (hereafter referred to S10F). Cell-free HSV-1 [F-strain (ATCC ID: VR-733) or HSV-1.VP16-GFP (recombinant HSV-1 expressing GFP conjugated to viral protein VP16) (La Boissiere et al., 2004) was obtained by scraping monolayers of virus-infected cells showing >80% cytopathic effect (CPE) in culture medium [DMEM/Ham’s F12 supplemented with 2% FBS and antibiotics (S2F)], followed by three successive freeze-thaw cycles and clarification at 1000 × *g* for 15 min (Ouwendijk et al., 2014). Cell-free VZV (clinical isolate EMC-1, passages 8 to 13) was obtained by scraping monolayers of virus-infected cells showing 30–50% CPE in PSGC buffer [PBS containing 5% (w/v) sucrose, 0.1% monosodium glutamate and 10% FBS (all from Sigma-Aldrich)], followed by sonication for 3 × 15 s and clarification for 15 min at 1,000 × *g* (Schmidt and Lennette, 1976; Harper et al., 1998). For mass-spectrometry experiments VZV preparations were subsequently concentrated using Lenti-X Concentrator (Clontech) according to the manufacturer’s instructions and resuspended in 1/10th of the original volume PSGC buffer (Sloutskin et al., 2013). HSV-1 and VZV stocks were stored at −80°C until use. Recombinant VZV.BAC-GFP ectopically expresses GFP, is not attenuated in cell culture, and was cultured on ARPE-19 cells as described (Zhang et al., 2008; Ouwendijk et al., 2014).

### Label-Free HSV-1 and VZV Samples for Mass-Spectrometry

ARPE-19 cells were plated at 2 × 10^5^ cells/well in 12-well plates and cultured overnight in S10F at 37°C in a CO2 incubator. Cells were washed twice with DMEM and infected with HSV-1 and VZV at MOI = 1 (2 × 10^5^ PFU/well) diluted in 600 μl DMEM. Alternatively, cells were infected with an equivalent volume of S2F or PSGC buffer diluted in DMEM as control for HSV-1 and VZV, referred to as “mock infection”. Infection efficiency was enhanced by spin-inoculation for 20 min at 1,000 x g, followed by incubation of cells at 37°C for 40 min. Infected cells were thoroughly washed with DMEM and 2 ml of S2F was added to each well (referred to as: *t* = 0 h). Mock-infected cells were harvested at 0 hr after infection, and virus-infected cells were harvested after the indicated intervals. Cells were scraped in ice-cold PBS, washed twice with 10 ml ice-cold PBS and cell pellets were stored at −80°C. Three independent experiments were performed.

### ^13^C_6_ L-Lysine- and ^13^C_6_ L-Arginine-Labeled VZV Samples for Mass-Spectrometry

SILAC was used to differentiate inoculum VZV proteins from newly synthesized viral proteins. ARPE-19 cells were cultured for five passages in S10F containing ^13^C_6_ L-Lysine and ^13^C_6_ L-Arginine according to the manufacturer’s instructions (Thermo Fisher Scientific). The labeling efficacy of cell cultures was checked using LC–MS and was larger than 95%. Labeled ARPE-19 cells were plated at 2.5 × 10^5^ cells/well in 12-well plates and cultured overnight in S10F containing ^13^C_6_ L-Lysine and ^13^C_6_ L-Arginine at 37°C in a CO2 incubator. VZV infection and harvesting of cells were performed as described above, with the following modifications: infection was performed in a 1:1 ratio (vol/vol) of DMEM and Ham’s F12 nutrient mixture containing ^13^C_6_ L-Lysine and ^13^C_6_ L-Arginine and maintained in S2F containing ^13^C_6_ L-Lysine and ^13^C_6_ L-Arginine. Three independent experiments were performed.

### In-Solution Digestion

Cell pellets were resuspended in 30 μl 0.2% RapiGest (Waters Corporation) in 50 mM NH_4_HCO_3_ and lysed by sonication for 2 min at 70% amplitude at a maximum temperature of 25°C (Branson Ultrasonics). Proteins were reduced with 10 mM dithiothreitol (DTT) at 60°C for 30 min, cooled to room temperature (RT), alkylated with 50 mM iodoacetamide in the dark for 30 min and digested overnight with 5 μl trypsin (0.1 μg/ul) (Promega). To inactivate trypsin and to degrade RapiGest, 4 μl of 5% TFA (Biosolve) were added and samples were incubated for 30 min at 37°C. Samples were centrifuged at maximum speed for 15 min at 4°C and the supernatants were transferred to LC vials and stored at 4°C until the measurements on the LC–MS were performed.

### LC–MS Measurements

Samples were measured on an LC-system and based on the integrated UV trace the injection volume for each sample was determined to ensure that an equivalent amount of ∼1 μg was loaded. Subsequently the determined injection volume of each sample was loaded on a nano-LC system (Ultimate 3000RS, Thermo Fisher Scientific). After preconcentration and washing of the sample on a C18 trap column (1 mm × 300 μm i.d., Thermo Fisher Scientific), sample was loaded onto a C18 column (PepMap C18, 75 mm ID × 500 mm, 2 μm particle and 100 Å pore size, Thermo Fisher Scientific) using a linear 90 min gradient (4–38% acetonitrile/H20; 0.1% formic acid) at a flow rate of 250 nL/min. The separation of the peptides was monitored by a UV detector (absorption at 214 nm). The nanoLC was coupled to a nanospray source of either an LTQ Orbitrap XL (HSV measurements) or a Orbitrap Fusion^TM^ Tribrid^TM^ mass spectrometer (VZV measurements) (Thermo Fisher Scientific). The following settings were used for the LTQ Orbitrap XL, a data-dependent acquisition method with a high-resolution survey scan from 400–1800 m/z was measured in the Orbitrap (target of automatic gain control = 106, resolution = 30,000 at 400 m/z, lock mass correction was activated to improve mass accuracy of the survey scan). On the basis of this full scan, the five most intensive ions were consecutively isolated (automatic gain control target set to 104 ions), fragmented via collision-activated dissociation (applying 35% normalized collision energy), and detected in the ion trap. Precursor masses within a tolerance range of ±5 ppm that were selected once for MS/MS were excluded for MS/MS fragmentation for 3 min or until the precursor intensity fell below a signal-to-noise ratio of 1.5 for more than five scans. Settings used for the Orbitrap Fusion^TM^ Tribrid^TM^ Full scan MS spectra (*m*/*z* 350–1600) in profile mode were acquired in the Orbitrap with a resolution of 120,000 after accumulation of an AGC target of 400,000. A top speed method with a maximum duty cycle of 3 s was used. In these 3 s the most intense peptide ions from the full scan in the Orbitrap were fragmented by collision induced dissociation (normalized collision energy 30%) and measured in the iontrap with a AGC target of 5,000. Maximum fill times were 100 ms for the full scans and 40 ms for the MS/MS scans. Precursor ion charge state screening was enabled and only charge states from 2 to 7 were selected for fragmentation. The dynamic exclusion was activated after the first time a precursor was selected for fragmentation and excluded for a period of 60 s using a relative mass window of 10 ppm. Lock mass correction was activated to improve mass accuracy of the survey scan.

### Mass–Spectrometry Data Analyses

From the raw data file of either the LTQ Orbitrap XL or the Orbitrap Fusion mass spectrometer, MS/MS spectra were extracted and converted into mgf files by using MSConvert of ProteoWizard (version 3.0.06245). All mgf files were analyzed using Mascot (version 2.3.02; the Matrix Science, London, United Kingdom). Mascot was used to perform database searches against the virus subset of the uniprot_sprot_2014_09 database; Virus species restriction; 11999 sequences) of the extracted MS/MS data. For the database search the following settings were used: a maximum of two miss cleavages, oxidation as a variable modification of methionine, carbamidomethylation as a fixed modification of cysteine and trypsin was set as enzyme. The SILAC measurements were also searched using two additional fixed modification ^13^C_6_ L-Lysine- and ^13^C_6_ L-Arginine to detect either the labeled peptides or the unlabeled peptides with search method without the modified lysine and arginine. A peptide mass tolerance of 10 ppm and a fragment mass tolerance of 0.5 Da were allowed. Scaffold software (version 4.4.3, Portland, OR) was used to summarize and filter MS/MS based peptides and protein identifications. Protein identifications were accepted if they could be established at greater than 95.0% peptide and protein probability and contained at least two identified peptides. Proteins that contained similar peptides and could not be differentiated based on MS/MS analysis alone were grouped. Using these criteria, Scaffold generated a list of identified proteins including the number of sequenced peptides and spectral counts for each sample.

In addition, the raw data files were loaded into the software package Progenesis QI (Version 4.0; Non-lineair Dynamics part of Waters). The data files were aligned and feature selection was performed. Subsequently, the identifications of the Scaffold analyses were loaded into Progenesis. Finally, a quantitative analyses was performed using the “relative quantification using non-conflicting peptides” quantification option in Progenesis. From the results an export file was created in which for each protein the obtained relative abundance in the different samples is displayed. Ingenuity Pathway Analysis software (Qiagen) was used to provide the cellular localization of host proteins.

### Statistical Analysis

To identify proteins that are differentially expressed over the course of infection, we performed differential protein expression analysis using limma (version 3.20.8, Bioconductor Biobase 2.24.0, R 3.1.3) (Team, 2014; Ritchie et al., 2015; Van Ooijen et al., 2018) on non-imputed protein data. Peptide data was log2-transformed and summarized to protein values using median polish (R 3.1.3, base package stats, medpolish). All MS-based protein expression levels are given on a log2 scale. Host and virus proteins were analyzed separately. We accounted for multiple testing by computing False Discovery Rates and indicating which proteins met FDR < 0.1 and FDR < 0.05 significance levels in the volcano plots. Principal component analysis on the protein data was also performed using R. Morpheus software^1^ was used to generate heatmaps and perform hierarchical cluster analysis. Hierarchical clustering was performed on absolute, log2-transformed data using the one minus Pearson correlation and average linkage method.

### Pathway Analysis

Pathway analysis was performed using the Database for Annotation, Visualization and Integrated Discovery (DAVID) version 6.8. Functional annotation was performed using differentially expressed host proteins (adjusted *p*-value < 0.05) and host proteins clustering with virus proteins as gene list and the total list of host proteins quantified in replicate experiments as background. Gene Ontology biological process, fold enrichment and modified Fishers’ exact test p-values were downloaded from the DAVID website (Huang Da et al., 2009a, b). The STRING database (Soday et al., 2019) was used to visualize protein interaction networks of differentially expressed host proteins conserved between HSV-1 and VZV. Host proteins differentially expressed by both viruses and differentially expressed host proteins with in the same Gene Ontology biological process were included.

### Kinetics of HSV-1 and VZV Replication in ARPE-19 Cells

To determine the kinetics of infectious virus production, ARPE-19 cells were infected with cell-free HSV-1 and VZV as described above in “Label-free HSV-1 and VZV samples for mass-spectrometry”. Infectious virus titers were determined, by conventional plaque assays using ARPE-19 cells, on supernatants (HSV-1) and infected cells (VZV) harvested at different time points post-infection. Two independent experiments were performed.

### Quantification of Virus DNA-to-PFU Ratio

Three independently generated cell-free HSV-1 and VZV stocks were used for DNA extraction and virus titration on ARPE-19 cells. Viral DNA was extracted using the QIAamp DNA Mini kit and analyzed by quantitative Taqman real-time PCR using primers and probes directed to HSV-1 US4 and VZV ORF38 as described previously (Van Velzen et al., 2013). Virus titers were determined by conventional plaque assay.

### Flow Cytometry

To analyze the effect of EGF signaling on HSV-1 and VZV replication, ARPE-19 cells were plated at 5 × 10^4^ cells/well in 48-well plates and cultured overnight in S10F at 37°C in a CO2 incubator. Cells were washed with DMEM and infected with HSV-1.VP16-GFP (10^2^ PFU) or cell-free VZV-BAC-GFP (2–3 × 10^3^ PFU) diluted in 250 μl DMEM per well and incubated at 37°C for 4 h. Virus inoculum was removed, cells were washed twice with DMEM and fresh S2F containing 0 ng/ml, 1 ng/ml, or 10 ng/ml recombinant human EGF (Peprotech) was added. Alternatively, S2F containing 0 μM, 6.1 μM, 12.5 μM, or 25 μM of the specific EGFR inhibitor AG 1478 (Abcam) was added. HSV-1-infected cells were harvested at 24, 32, and 48 hpi. VZV-infected cells were harvested at 24, 48, and 72 hpi. Cells were washed with FACS buffer (PBS containing 0.05% bovine serum albumin and 2 mM EDTA), fixed for 15 min in 4% paraformaldehyde (PFA) in PBS, washed and resuspended in FACS buffer, and GFP expression was measured on a BD FACS Lyric (BD biosciences). Experiments were performed in triplicate and at least two independent experiments were performed.

To analyze the effect of productive HSV-1 and VZV infection on surface expression of PLAA and LOX, respectively, ARPE-19 cells were infected as described above. HSV-1 infected cells were harvested at 24 hpi and VZV-infected cells at 72 hpi. Cells were washed with FACS buffer, blocked for 30 min using 5% normal goat serum diluted in FACS buffer and stained with primary antibodies diluted in FACS buffer. After washing, cells were incubated with secondary antibody diluted in FACS buffer, washed with FACS buffer, PFA-fixed and analyzed on a BD FACS Lyric. Experiments were performed in triplicate and two independent experiments were performed.

For confirmation of HSV-1 protein expression, ARPE-19 cells were plated at 5 × 10^5^ cells/well in 6-well plates and cultured overnight in S10F at 37°C in a CO2 incubator. Cells were washed twice with DMEM and infected with HSV-1 F-strain at multiplicity of infection (MOI) = 1 diluted in 1 ml DMEM, spin-inoculated for 20 min at 1,000 × *g* and incubated at 37°C for 40 min. Cells were thoroughly washed with DMEM and 2ml of S2F was added to each well (referred to as: *t* = 0 hr). Mock-infected cells were harvested at 0 h after infection, and virus-infected cells were harvested after the indicated intervals. Cells were washed with FACS buffer, fixed and permeabilized using Cytofix/Cytoperm (BD Biosciences) blocked using 5% goat serum (Sigma Aldrich) diluted in PermWash solution (BD Biosciences). Cells were stained with primary antibody diluted in PermWash, washed with PermWash and incubated with secondary antibody diluted in PermWash. After a final wash, cells were resuspended in FACS buffer for measurement on a BD FACS Canto II (BD biosciences). Two independent experiments were performed.

### Cytotoxicity Assay

To analyze the effect of AG1478 treatment on viability of ARPE-19 cells, 5 × 10^4^ cells/well were plated in 48-well plates and cultured overnight in S10F at 37°C in a CO2 incubator. Cells were washed with DMEM and incubated with S2F containing 0, 6.1, 12.5, 25, or 50 μM AG1478. Supernatants were harvested at 24, 48, and 72 h and stored at −20°C until analysis. Lactate dehydrogenase (LDH) levels in supernatant were analyzed by ELISA using the PierceTM LDH Cytotoxicity Assay Kit (Thermo Fisher Scientific), according to the manufacturer’s instructions. Three independent experiments were performed.

### Western Blotting

For kinetic analysis of VZV protein expression cells were infected and processed as described above in “Label-free HSV-1 and VZV samples for mass-spectrometry.”

For confirmation of HSV-1-induced SPARC downregulation, confluent monolayers of ARPE-19 cells grown in 25 cm^2^ flasks were infected with HSV-1.VP16-GFP (MOI = 1) for 24 h. To analyze EGFR and phosphorylated EGFR expression, confluent monolayers of ARPE-19 cells were grown in 25 cm^2^ flasks and infected with HSV-1.VP16-GFP (MOI = 1) for 24 h, VZV.BAC-GFP-infected ARPE-19 cells (ratio of one VZV-infected cell to eight uninfected cells) for 72 h or left infected. In some experiments, cells were stimulated with recombinant human EGF (1 or 10 ng/ml diluted in S10F) for 30 min at 37°C before cell lysis.

Cells were harvested by scraping in ice cold PBS, pelleted by centrifugation for 5 min at 300 × *g* at 4°C and lysed in 100 μl RIPA buffer (150 mM NaCl, 1% NP40, 0.1% SDS, 0.5% Na-deoxycholate and 50 mM TrisHCl pH = 8.0) containing protease and phosphatase inhibitors (Roche) by rotating for 30 min at 4°C. Cell lysates were centrifuged at 14,000 × *g* for 5 min and supernatants were used for protein quantification (Pierce BCA Protein Assay Kit; Thermo Fisher Scientific) and stored at −80°C. Total cell lysates (30 μg) were separated by SDS-Page on 4–20% or 10% polyacrylamide gels (Bio-Rad) and transferred to Immobilon-FL PVDF membranes (Merck). Membranes were blocked for 1 h in 10% milk powder/PBS at RT, stained with primary antibodies diluted in 5% milk powder/TBS-T (150 mM NaCl, 10 mM Tris pH 8.0) overnight at 4°C (HSV proteins) or 90 min at RT (host and VZV proteins) and incubated the secondary antibodies for 60 min at RT. Membranes were analyzed using LI-COR Odyssey Infrared Imaging System and Odyssey 3.0 software.

### Confocal Microscopy

ARPE-19 cells grown on glass coverslips were infected with cell-free HSV-1.VP16-GFP (MOI = 0.05–0.1) or VZV.BAC-GFP (MOI = 0.05) for 24 h, PFA-fixed for 15 min at RT and washed with PBS. Cells were permeabilized with 0.1% (v/v) Triton X-100 in PBS for 10 min, blocked for 30 min using 5% normal goat serum diluted in PBS and incubated with primary antibodies diluted in PBS containing 0.1% bovine serum albumin (BSA) for 1 h at RT. Cells were washed with PBS, incubated with secondary antibodies diluted in 0.1%BSA/PBS for 1 h at RT, washed, incubated with 20 μM Hoechst 33342 solution (Thermo Fisher Scientific), washed twice with PBS and mounted in Prolong Diamond antifade reagent (Thermo Fisher Scientific). Cells were analyzed using a Zeiss LSM 700 confocal laser scanning microscope. ZEN 2010 software (Zeiss) was used to adjust brightness and contrast.

## Results

### Temporal Viromic Analysis of Productive HSV-1 Infection

**FIGURE 1:**
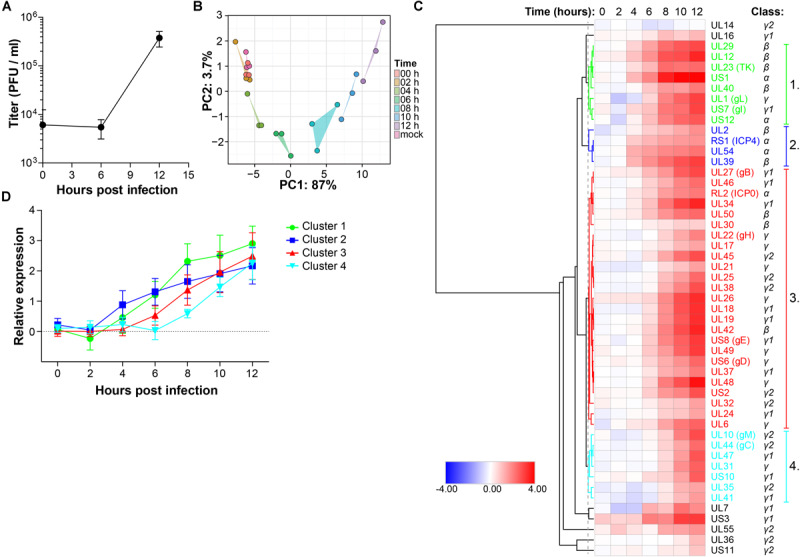
Temporal analysis of the HSV-1 proteome during productive infection of ARPE-19 cells by mass spectrometry. **(A)** Infectious virus titer in supernatant of HSV-1-infected ARPE-19 cells (F-strain, MOI = 1) at the indicated time points. Data shown indicate average ± SD of *n* = 2 independent experiments. **(B–D)** HSV-1-infected ARPE-19 cells (F-strain, MOI = 1) were analyzed by MS. Three independent experiments were performed. **(B)** Principal component analysis of MS results, with the first and second principal components (PC1, PC2) and their corresponding variances depicted on the *x*- and *y*-axis, respectively. **(C)** Heatmap showing average log_2_-fold change in HSV-1 protein expression. Major clusters of viral proteins are indicated by number and font color. Reported kinetic classes of HSV-1 proteins are indicated. **(D)** Relative protein expression (average ± SD log_2_-fold change) of viral proteins from each cluster.

Retinal pigment epithelial cells are a clinically relevant and highly permissive cell type for both HSV-1 and VZV infection (Ouwendijk et al., 2014), facilitating a proteomic analysis of productive infection with both αHHV. In our experimental setting, infectious HSV-1 virions are produced at 12 h post-infection (hpi) in ARPE-19 cells (Figure 1A) and pilot mass-spectrometry (MS) analysis showed that similar numbers of HSV-1 proteins could be identified at 12 and 24 hpi (Supplementary Figure S1A). Therefore, we performed temporal viromic analysis of HSV-1-infected ARPE-19 cells over a 12-h period, using 2-h intervals, by MS. In total 51 of 73 (70%) canonical HSV-1 proteins included in the UniProt database (The Uniprot Consortium, 2017) were consistently detected in three independent experiments (Supplementary Table S1). Post-translational modifications were identified in 14 HSV-1 proteins at 12 hpi (Supplementary Table S2). Principal component analysis (PCA) of detected HSV-1 proteins showed a high degree of reproducibility among experiments, with mock, 0 and 2 hpi samples clustering closely together and the remaining samples forming distinct clusters per time point (Figure 1B). Abundance of HSV-1 proteins increased in time from 0 to 12 h (Figure 1C) and no decline in α or β gene protein quantities was observed at later times post-infection. Graphs for each individual HSV-1 protein are shown in Supplementary Figure S2.

Next, hierarchical cluster analysis was used to analyze the temporal pattern of viral protein expression during productive infection of ARPE-19 cells (Figure 1C). Four major clusters were identified. HSV-1 proteins in clusters 1 and 2 were expressed relatively early after infection, followed by those in cluster 3 and lastly viral proteins in cluster 4 (Figure 1D). Consistent with their reported kinetic class (Roizman et al., 2013), clusters 1 and 2 mainly contained HSV proteins encoded by α- and β-genes, whereas viral proteins in clusters 3 and 4 were mostly encoded by γ-genes (Figure 1C and Supplementary Figure S1B). Similar findings were obtained using an alternative approach to determine the kinetics of HSV-1 protein expression, based on the time points when quantified viral proteins were first significantly (adjusted *p*-value < 0.05) expressed above baseline normalized signal intensities of MS spectra in mock-infected cells (Supplementary Figures S1C,D).

**FIGURE 2:**
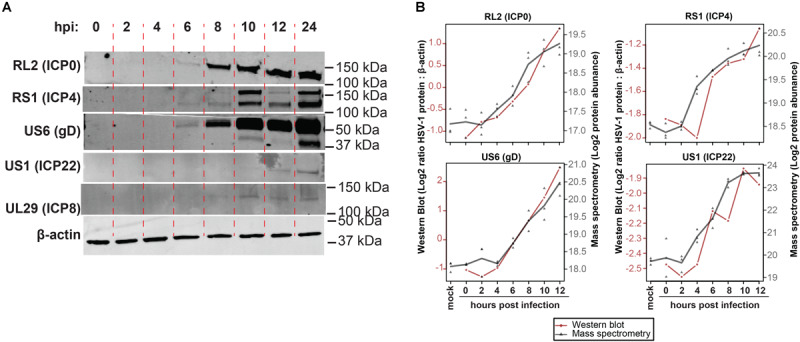
Temporal analysis of selected HSV-1 proteins during productive infection of ARPE-19 cells by western blotting. **(A)** HSV-1-infected ARPE-19 cells (F-strain, MOI = 1) were analyzed by WB using antibodies directed to the indicated five HSV-1 proteins. Two independent experiments were performed. hpi: hours post-infection. **(B)** Overlay of WB and MS results, with the different time points indicated on the *x*-axis, western blot normalized protein abundance (ratio average HSV-1 protein: β-actin protein signal intensity) on the left *y*-axis, and mass spectrometry log_2_-transformed protein abundances on the right *y*-axis. WB data: red line and circles indicate mean WB normalized protein abundances (RL2 and US6: *n* = 3 independent experiments; RS1 and US1: *n* = 2 independent experiments). MS data: gray triangles indicate individual values (*n* = 3 independent experiments) and gray line indicates mean protein abundance.

To confirm MS results, expression of five representative viral proteins in HSV-1 infected ARPE-19 cells was determined by western blotting (WB) (Figure 2A). HSV-1 proteins were selected based on their kinetic class, MS expression pattern and availability of specific antibodies applicable for WB: RL2 (ICP0; α, ≥8 hpi), RS1 (ICP4; α, significantly detected at ≥4 hpi in MS results), US6 (gD; γ, ≥8 hpi), US1 (ICP22; α ≥ 8 hpi), UL29 (ICP8; β, ≥6 hpi). WB analysis consistently detected ICP0, ICP4 and gD expression from 4 hpi, UL29 protein from eight hpi and US1 protein from 12 hpi onward (Figure 2A). Similar expression patterns of ICP0, ICP4 and gD were observed by MS and WB (Figure 2B and Supplementary Figure S3), with slightly delayed detection of US1 and UL29 proteins by WB compared to MS. Overall, unbiased HSV-1 proteome-wide MS analysis and subsequent WB of productively HSV-1-infected ARPE-19 cells support temporal expression of HSV-1 proteins, generally compatible with reported kinetic class of their corresponding mRNAs (Roizman et al., 2013).

### Temporal Viromic Analysis of Productive VZV Infection

**FIGURE 3:**
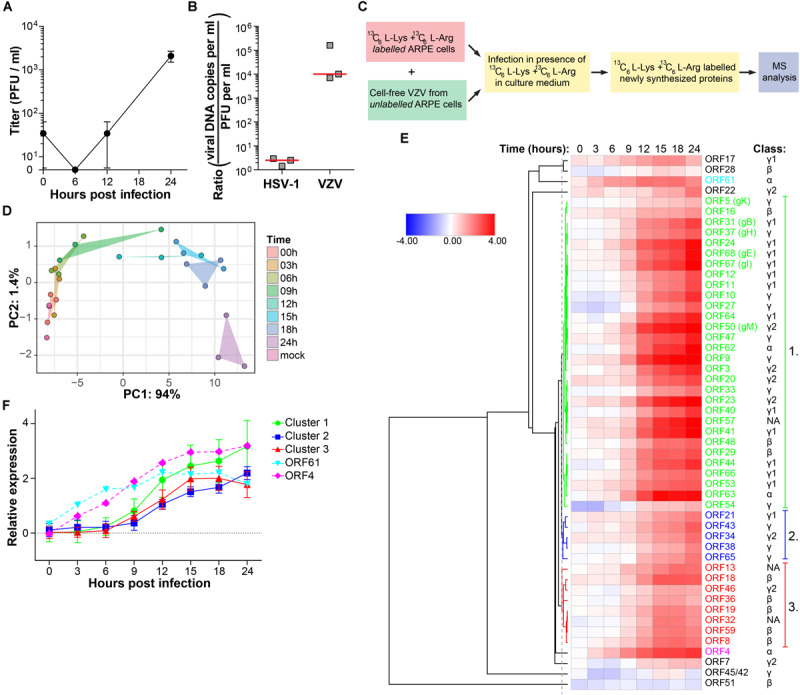
Temporal analysis of the VZV proteome during productive infection of ARPE-19 cells by mass spectrometry. **(A)** Cell-free VZV titers obtained from VZV-infected ARPE-19 cells (cell free VZV EMC-1 strain, MOI = 1) at the indicated time points after infection. Data shown indicate average ± SD of *n* = 2 independent experiments. **(B)** Enumeration of the viral DNA – to – PFU ratio in ARPE-19 cells. Three independently generated cell-free HSV-1 and VZV stocks were used for DNA extraction and virus titration on ARPE-19 cells. Viral DNA load and infectious titers were determined by qPCR and plaque assay, respectively. Horizontal line indicates median. **(C)** ^13^C_6_-L-Lysine and ^13^C_6_-L-Arginine labeled ARPE-19 cells were infected with cell-free VZV (strain EMC-1, MOI = 1), in the presence of ^13^C_6_-L-Lysine and ^13^C_6_-L-Arginine to label newly synthesized proteins, and analyzed by MS. Three independent experiments were performed. **(D)** Principal component analysis of MS results, with PC1 and PC2 and their corresponding variances depicted on the *x*- and *y*-axis, respectively. **(E)** Heatmap showing average log_2_-fold change in VZV protein expression. ORF4, ORF61 and three major clusters of viral proteins are indicated by number and font color. Putative kinetic classes of VZV proteins, based on the kinetic class of their HSV-1 homologs, are indicated. **(F)** Relative protein expression (average ± SD log_2_-fold change) of viral proteins from each cluster, as well as ORFs 4 and 61.

In our experimental setting, infectious VZV virions were produced at 24 hpi, but not 12 hpi in ARPE-19 cells (Figure 3A). To determine whether VZV proteins were expressed in a temporally coordinated fashion we analyzed VZV-infected ARPE-19 cells at multiple time points after infection. However, 32 VZV proteins were detected already at 0 hpi, which increased to 38 VZV proteins at 12 hpi and 41 at 24 hpi (Supplementary Figures S4A,B). Because most VZV proteins detected at 0 hpi were structural proteins, these data were most likely caused by the extremely high number of defective virus particles produced by VZV-infected cells: particle-to-plaque-forming unit (PFU) ratio of 40,000: 1 compared to a particle-to-PFU ratio of 10:1 for HSV-1 (Watson et al., 1963; Carpenter et al., 2009). We determined the viral genome equivalent copy-to-PFU ratio, as a conservative surrogate marker for the particle-to-PFU ratio (Carpenter et al., 2009), to confirm that VZV has a much higher viral DNA-to-PFU ratio (median 1.0 × 10^4^, range 7.0 × 10^3^ – 1.6 × 10^5^) compared to HSV-1 (median 2.5, range 1.4–3.0) in ARPE-19 cells (Figure 3B).

Therefore, we used a modified stable isotope labeling by amino acids in cell culture (SILAC) approach to discriminate virus inoculum proteins from newly produced proteins within the VZV-infected ARPE-19 cells (Figure 3C). The sensitivity of the SILAC-based MS approach was validated by determining the kinetics of VZV protein expression at 6, 12, and 24 hpi (Supplementary Figure S4C). Because infectious VZV could only be recovered from infected ARPE-19 cells starting at 24 hpi and the number of VZV proteins detected by MS increased from 12 to 24 hpi (Supplementary Figure S4C), we performed temporal viromic MS analysis of VZV protein expression in SILAC-labeled VZV-infected ARPE-19 cells over a 24-h period, using 3-h intervals and in three independent experiments. In total 51 of 69 (74%) canonical VZV proteins were consistently detected between biological triplicates at 24 hpi (Supplementary Table S3). Post-translational modifications were identified in 8 VZV proteins at 24 hpi (Supplementary Table S4). PCA of VZV proteins, showing larger variability between experiments compared to HSV-1 (Figure 1B), revealed that samples obtained after 6 hpi clustered distinctly from the cluster containing mock and 0 hpi samples (Figure 3D). Clusters overlapped for samples obtained at 3 – 6 – 9 hpi and 12 – 15 – 18 hpi, whereas the 24 hpi sample clustered separately (Figure 3D). Abundance of all VZV proteins increased in time from 0 to 24 hpi (Figure 3E) and no decline in VZV α or β gene protein quantities was observed at later times post infection. Graphs for individual viral proteins are provided in Supplementary Figure S5.

The temporal pattern of VZV protein expression was analyzed by hierarchical cluster analysis (Figure 3E). Three major clusters were identified: Cluster one is composed of 29 VZV proteins that were expressed before those of the smaller cluster two (five VZV proteins) and cluster three (eight VZV proteins) (Figure 3F). Notably, two VZV proteins, ORF4 and ORF61, were abundantly expressed at 3–6 hpi already, prior to viral proteins of cluster 1 (Figure 3F). Again, similar patterns of viral protein expression were obtained when we determined the time points when the quantified viral proteins were first significantly (adjusted *p*-value < 0.05) expressed above baseline signal intensities of MS spectra in mock-infected cells (Supplementary Figures 4E,F).

**FIGURE 4:**
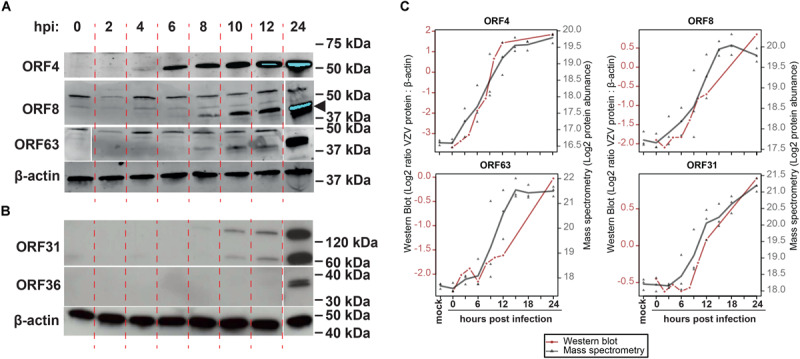
Temporal analysis of selected VZV proteins during productive infection of ARPE-19 cells by western blotting. **(A,B)** VZV-infected ARPE-19 cells (EMC-1, MOI = 1) were analyzed by WB using antibodies directed to the indicated five VZV proteins and human β-actin protein. Protein signal was visualized using fluorescence **(A)** and chemiluminescence **(B)**. Two independent experiments were performed. Arrowhead indicates specific band corresponding to ORF8. **(C)** Overlay of WB and MS results, with the different time points indicated on the *x*-axis, western blot normalized protein abundance (ratio average VZV protein: β-actin protein signal intensity) on the left *y*-axis, and mass spectrometry log_2_-transformed protein abundances on the right *y*-axis. WB data: red line and circles indicate mean WB normalized protein abundances (*n* = 2 independent experiments). MS data: gray triangles indicate individual values (*n* = 3 independent experiments) and gray line indicates mean protein abundance.

To confirm MS results, the expression of five representative viral proteins was determined in VZV-infected ARPE-19 cells by WB. VZV proteins were selected based on their putative kinetic class, the observed MS expression pattern, availability of specific antibodies applicable for WB, and absence of detectable protein levels at 0 hpi by WB: ORF4 (α, significantly detected at ≥9 hpi), ORF8 (β, ≥12 hpi), ORF31 (gB; γ, ≥12 hpi), ORF36 (β, ≥12 hpi) and ORF63 (α, ≥12 hpi) (Figure 4 and Supplementary Figure S6). Although most VZV proteins were detected slightly earlier by WB compared to MS (Figures 4A,B), overall expression patterns of ORF4, ORF8, ORF31 (gB) and ORF63 were similar between both methodologies (Figure 4C). Thus, the unbiased VZV proteome-wide MS analysis and subsequent confirmation of selected viral proteins by WB provide evidence for temporal, but not necessarily coordinated, expression of VZV proteins during productive infection.

### Temporal Analysis of Host Proteins During Productive HSV-1 Infection

**FIGURE 5:**
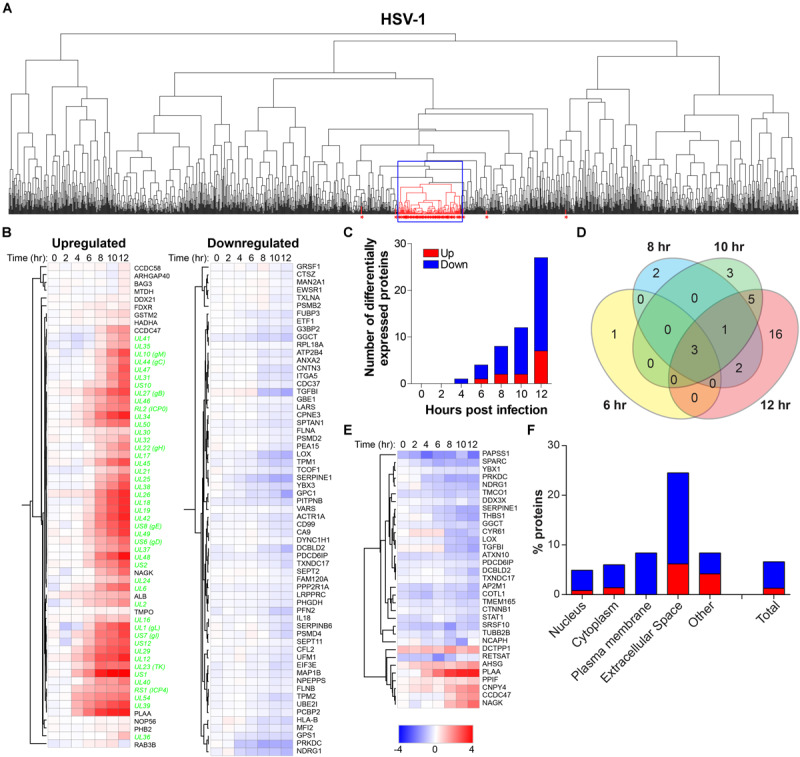
Temporal analysis of the host proteome during productive HSV-1 infection of ARPE-19 cells. Analysis of the host proteome in HSV-1-infected ARPE-19 cells (Figure 1) by MS. **(A)** Hierarchical cluster analysis of the virus and host proteins in HSV-1-infected cells. Viral proteins are indicated by red asterisks. Box indicates cluster containing majority of HSV-1 proteins. **(B)** Heatmap showing log2-fold change of up- and downregulated host (black font) proteins that clustered with virus proteins (green font) (box in panel A). **(C)** Number of differentially expressed host proteins in HSV-1-infected cells compared to mock-infected cells (adjusted *p*-value < 0.05). **(D)** Venn diagram indicating the number of significant differentially expressed host proteins at 6, 8, 10, and 12 hpi and the overlap between each set of proteins. **(E)** Heatmap showing log_2_-fold change of significant differentially expressed host proteins. **(F)** Cellular localization of host proteins that are up- and down-regulated during HSV-1 infection.

To determine the effect of productive HSV-1 infection on the host cell, temporal changes in host protein expression in HSV-1-infected ARPE-19 cells were analyzed. MS consistently detected 1,526 human proteins in all three independent experiments (Supplementary Table S5). Initially, we performed hierarchical cluster analysis to identify host proteins that clustered with virus proteins, indicative of possible co-regulated expression of host and HSV-1 proteins (Figure 5A). The expression pattern of 76 host proteins, encompassing 16 upregulate and 63 downregulated proteins, correlated with HSV-1 protein expression (Figure 5B). Additional host proteins affected by HSV-1 infection were identified by comparison of protein abundance in HSV-1-infected and mock-infected cells. We identified 33 statistically differentially expressed host proteins (DEPs), including seven upregulated and 26 downregulated proteins compared to mock-infected cells. Number of DEPs increased over time, with most DEPs remaining consistently up- or downregulated (Figures 5C,E). Combined, hierarchical clustering and quantitative protein level analyses identified 100 host proteins of interest (POIs), including 20 upregulated and 80 downregulated POIs (Supplementary Table S6). These data are consistent with previously reported downregulation of the DNA-dependent protein kinase catalytic subunit (PRKDC) (Lees-Miller et al., 1996), thrombospondin-1 (THBS1) (Choudhary et al., 2005), plasminogen activator inhibitor 1 (SERPINE-1) (Bok et al., 1993) and STAT1 (Chee and Roizman, 2004; Johnson and Knipe, 2010) by HSV-1 in infected cells. Notably, HSV-1 infection had the most pronounced impact on proteins expressed in the plasma membrane (all downregulated) and extracellular space (both up- and downregulated) (Figure 5F).

**FIGURE 6:**
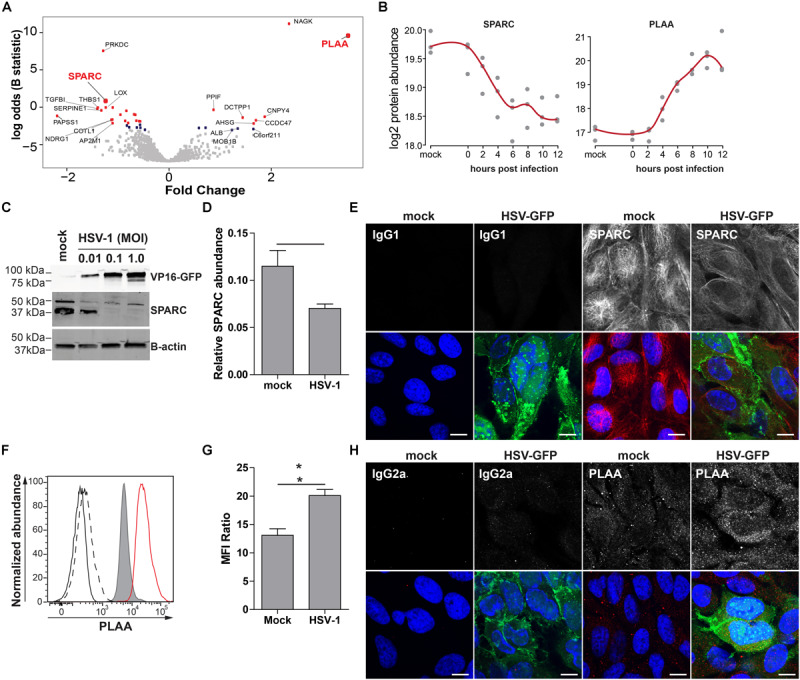
Confirmation of up- and downregulated expression of selected host proteins during productive HSV-1 infection of ARPE-19 cells. **(A)** Volcano plot of host protein expression at 12 hpi compared to mock-infected cells. Log_2_-fold change protein expression is indicated on the *x*-axis and significance (log odds) on the *y*-axis. The 10 most significant differentially expressed proteins are indicated (gray squares: FDR > 0.1; blue squares: FDR < 0.1; red squares: FDR < 0.05). Bold red font: proteins selected for confirmation. **(B)** Log_2_-transformed SPARC and PLAA protein abundance during HSV-1 infection. **(C–H)** HSV-1.VP16-GFP (HSV-1-GFP)-infected ARPE-19 cells were analyzed at 24 hpi. **(C)** Protein lysates were probed with antibodies specific to GFP, SPARC and β-actin. **(D)** Signal intensity of SPARC and β-actin were quantified and the average ratios SPARC/β-actin (*n* = 4 experiments) ± SEM are shown. **(E)** Mock- and HSV-1-GFP-infected (green) cells were stained for SPARC (red). **(F)** Histogram showing PLAA expression in mock-infected (filled, gray) and HSV-1-infected cells (red line) by flow cytometry. Fluorescence minus one (FMO) controls are shown for uninfected (black line) and HSV-1-infected cells (dashed black line). **(G)** Median fluorescent intensity (MFI) ratio (PLAA-stained/FMO control) for HSV-1-infected and mock-infected cells (*n* = 3 experiments) ± SEM are shown. **(H)** Confocal microscopy pictures of uninfected and HSV-1-GFP-infected (green) cells stained for PLAA. **(E,H)** Representative image for n = 3 independent experiments; Scale bar: 10 μm; Nuclei were stained with Hoechst (blue). **(D,G)** * *p* < 0.05 by paired Student’s t-test.

To confirm MS results, we analyzed the expression of phospholipase A-2-acitivating protein (PLAA) and secreted protein acidic and rich in cysteine protein (SPARC/osteonectin) in mock- and HSV-1-infected ARPE-19 cells. PLAA (upregulated by HSV-1) and SPARC (downregulated by HSV-1) were selected based on fold change protein expression (Figures 6A,B), statistical significance, availability of antibodies, and absence of prior literature reporting that their expression is affected by HSV-1. PLAA is a 87 kDa protein that locates to the cytoplasm and nucleus, and plays a role in protein ubiquitination, sorting and degradation (Papadopoulos et al., 2017). SPARC, a ∼40 kDa protein that locates to the cytoplasm and extracellular matrix, is a collagen-binding matricellular protein that regulates cell growth through its interactions with the extracellular matrix and cytokines (Bradshaw and Sage, 2001). Consistent with MS results, WB and confocal microscopy analysis of HSV-1 infected ARPE-19 cells stained with antibodies directed to SPARC, PLAA or appropriate isotype control antibodies showed that HSV-1 significantly downregulated SPARC expression (Figures 6C–E and Supplementary Figure S7A). Contrastingly, PLAA expression was significantly upregulated on HSV-1-infected ARPE-19 cells (Figures 6F–H and Supplementary Figure S7B).

Next, the identified host POIs were used to identify host cell processes most severely affected by HSV-1 infection using DAVID Bioinformatics Resources (Huang Da et al., 2009a, b; Table 1). Upregulated host proteins were involved in regulation of apoptosis, whereas downregulated proteins were mainly involved in organization of the extracellular matrix, cell adhesion, transcription and growth factor receptor signaling.

**TABLE 1:** Gene ontology enrichment analysis of host proteins affected by HSV-1 infection^a^.

**GO term (biological process)^b^**	***p*-value**	**Fold enrichment**	**Host proteins^c^**
**Upregulated proteins**			
GO:0043066∼negative regulation of apoptotic process	6.66E-03	5.92	ALB, BAG3, MTDH, PHB2, PPIF
**Downregulated proteins**			
GO:0030198∼extracellular matrix organization	3.4E-04	6.70	CCN1, ITGA5, LOX, SERPINE1, SPARC, TGFBI, THBS1
GO:0007155∼cell adhesion	8.7E-03	3.72	CCN1, CD99, CNTN3, CTNNB1, ITGA5, THBS1, TGFBI
GO:0045944∼positive regulation of transcription from RNA polymerase II promoter	1.0E-02	2.87	CCN1, CTNNB1, DDX3X, FUBP3, IL18, PRKDC, SERPINE1, STAT1, YBX1
GO:0071363∼cellular response to growth factor stimulus	1.6E-02	6.96	CTNNB1, CPNE3, SPARC, THBS1
GO:0042060∼wound healing	1.6E-02	6.96	DCBLD2, LOX, SPARC, TPM1
GO:0001701∼*in utero* embryonic development	2.4E-02	4.35	CTNNB1, MAN2A1, TPM1, YBX1, YBX3
GO:0016525∼negative regulation of angiogenesis	2.4E-02	11.49	SPARC, STAT1, THBS1
GO:0001525∼angiogenesis	2.7E-02	4.16	ANXA2, IL18, ITGA5, SERPINE1, TGFBI
GO:0001937∼negative regulation of endothelial cell proliferation	3.4E-02	9.58	SPARC, STAT1, THBS1

*^*a*^Gene ontology enrichment analysis of host proteins of interest (Supplementary Table S6) using DAVID Bioinformatics Resources. ^*b*^Enriched gene ontology (GO) biologically processes (Modified Fisher’s exact test: *p* < 0.05). ^*c*^Gene names of differentially expressed host proteins categorized into the GO biological process indicated.*

### Temporal Analysis of Host Proteins During Productive VZV Infection

**FIGURE 7:**
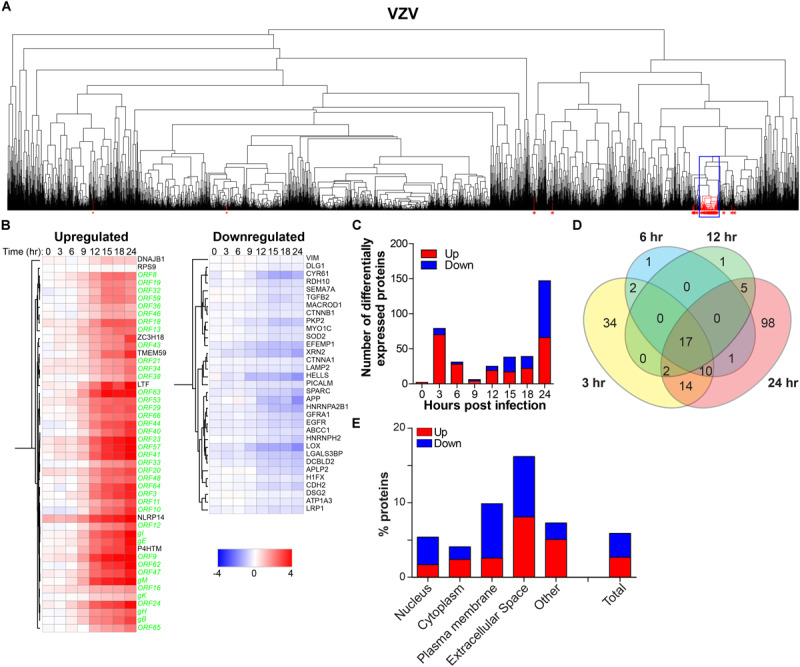
Temporal analysis of the host proteome during productive VZV infection of ARPE-19 cells. Analysis of the host proteome in VZV-infected ARPE-19 cells (Figure 3) by MS. **(A)** Hierarchical cluster analysis of the virus and host proteins in VZV-infected cells. Viral proteins are indicated by red asterisks. Box indicates cluster containing majority of VZV proteins. **(B)** Heatmap showing log2-fold change of up- and downregulated host (black font) proteins that clustered with virus proteins (green font) [box in panel **(A)**]. **(C)** Number of differentially expressed host proteins in VZV-infected cells compared to mock-infected cells (adjusted *p*-value < 0.05). **(D)** Venn diagram indicating the number of significant differentially expressed host proteins at 3, 6, 12, and 24 hpi and the overlap between each set of proteins. **(E)** Cellular localization of host proteins that are up- and down-regulated during VZV infection.

To determine the effect of productive VZV infection on the host cell, temporal changes in host protein expression were analyzed in the VZV-infected SILAC-labeled ARPE-19 cells. MS consistently detected 3,714 human proteins in all three independent experiments (Supplementary Table S7). Hierarchical cluster analyses identified 40 host proteins, including seven upregulated and 33 downregulated proteins, that correlated with the expression of VZV proteins (Figures 7A,B). Additionally, abundance of 200 host proteins was statistically significantly affected by VZV infection, encompassing 99 upregulated and 101 downregulated proteins compared to mock-infected ARPE-19 cells (Supplementary Table S8). VZV infection induced a rapid transient burst in DEPs (*n* = 79, including 70 upregulated and nine downregulated proteins) at 3 hpi, that subsequently declined to only seven DEPs at nine hpi (Figures 7C,D), which was not observed for HSV-1 infection (Figure 5). At 24 hpi, number of DEPs steadily increased to 147 (encompassing 66 upregulated and 81 downregulated proteins) (Figures 7C,D). Combined, hierarchical clustering and quantitative protein level analyses identified 219 host POIs, including 100 upregulated and 119 downregulated POIs. Our results are consistent with previously reported upregulation of elongation factor 1-alpha (EEF1A2) (Zuranski et al., 2005) and cyclin-B1 (CCNB1) (Liu and Cohen, 2013) in VZV-infected cells. Similar to HSV-1 infection (Figure 5F), VZV infection also had the most severe impact on proteins expressed in the extracellular space and plasma membrane (Figure 7E).

**FIGURE 8:**
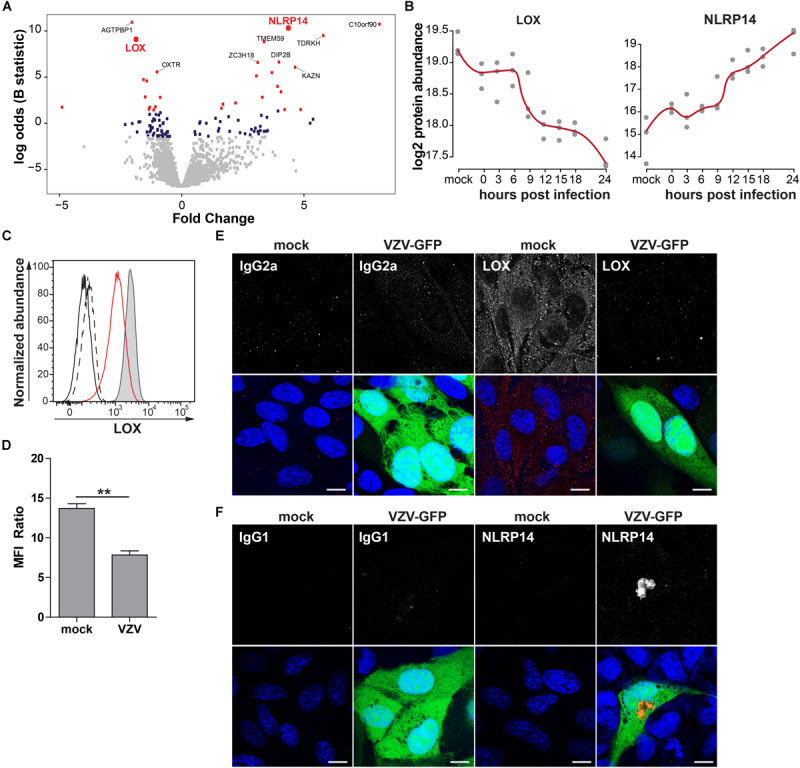
Confirmation of up- and downregulated expression of selected host proteins during productive VZV infection of ARPE-19 cells. **(A)** Volcano plot of host protein expression at 3 and 24 hpi compared to mock-infected cells. Log_2_-fold change protein expression is indicated on the *x*-axis and significance (log odds) on the *y*-axis. The 10 most significant differentially expressed proteins are indicated (gray squares: FDR > 0.05; blue squares: FDR < 0.05; red squares: FDR < 0.01). Bold red font: proteins selected for confirmation. **(B)** Log_2_-transformed LOX and NLRP14 protein abundance during VZV infection. **(C)** Histogram showing LOX expression in uninfected (filled, gray) and cell-free VZV-infected ARPE-19 cells (red line) by flow cytometry. Fluorescence minus one (FMO) controls are shown for uninfected (black line) and VZV-infected cells (dashed black line). **(D)** Median fluorescent intensity (MFI) ratio (LOX-stained/FMO control) for VZV-infected and mock-infected cells (n = 3 experiments). **(E,F)** Confocal microscopy pictures of mock-infected or cell-free VZV.BAC-GFP (VZV-GFP; green) infected ARPE-19 cells stained for LOX **(E)** and NLRP14 **(F)** (red). Representative image for *n* = 3 independent experiments; Scale bar: 10 μm; Nuclei were counterstained with Hoechst (blue).

To confirm MS results, expression of NACHT, LRR and PYD domains-containing protein 14 (NLRP14; upregulated) and protein-lysine 6-oxidase (LOX; downregulated) was analyzed by immunocytology and flow cytometry on mock- and VZV-infected ARPE-19 cells. LOX, a copper-containing amine oxidase of 47 kDa located in the endoplasmic reticulum (Guo et al., 2016), and NLRP14, a cytoplasmic NOD-like receptor of ±125 kDa (Abe et al., 2017), were selected based on fold change protein expression (Figures 8A,B), statistical significance, availability of antibodies, and absence of prior literature reporting that their expression is affected by VZV infection. Consistent with MS data, VZV infection downregulated LOX and upregulated NLRP14 expression in ARPE-19 cells (Figures 8C,F and Supplementary Figure S8).

Identified POIs were used to identify cellular processes affected by VZV infection. Upregulated proteins were mainly involved in cell division and cilium function, whereas downregulated proteins functioned predominantly in (m)RNA processing, extracellular matrix organization and cell adhesion (Table 2). Interestingly, host proteins induced early after VZV infection at three hpi were mainly involved in extracellular matrix remodeling, with downregulated proteins involved in RNA processing (Table 3). Overall, productive VZV infection appears to induce a cellular response that affects especially extracellular matrix organization, while at later stages after infection, VZV replication more severely impacts ECM composition, proliferation and cellular transcription/RNA processing.

**TABLE 2:** Gene ontology enrichment analysis of host proteins affected by VZV infection^a^.

**GO term (biological process)^b^**	***p*-value**	**Fold enrichment**	**Host proteins^c^**
**Upregulated proteins**			
GO:0042384∼cilium assembly	5.39E-04	8.9	ABCC4, C10orf90, EHD1, PCM1, RAB23, WDR19
GO:0051301∼cell division	5.77E-04	4.7	AURKA, BRCC3, CCNB1, CDC23, KIFC1, MAPRE2, NEK2, USP16, ZFYVE19,
GO:0030198∼extracellular matrix organization	6.47E-04	6.6	COL4A1, COL4A2, COL5A2, FBN1, FN1, GFAP, HSPG2
GO:0060271∼cilium morphogenesis	6.15E-03	6.8	EHD1, PCM1, RAB23, RO60, WDR19
GO:0000086∼G2/M transition of mitotic cell cycle	6.31E-03	6.7	AKAP9, AURKA, CCNB1, NEK2, PCM
GO:0031145∼anaphase-promoting complex-dependent catabolic process	8.78E-03	9.3	AURKA, CCNB1, CDC23, PSMD8
GO:0007067∼mitotic nuclear division	1.07E-02	4.5	AURKA, BRCC3, CDC23, MAPRE2, NEK2, USP16
GO:0070537∼histone H2A K63-linked deubiquitination	1.60E-02	123.0	BRCC3, USP16
GO:0007080∼mitotic metaphase plate congression	1.68E-02	15.0	CCNB1, CDC23, KIFC1
GO:0002576∼platelet degranulation	1.79E-02	7.2	ABCC4, AHSG, APOA1, FN1
**Downregulated proteins**			
GO:0006396∼RNA processing	5.13E-05	10.5	DDX17, DHX36, HNRNPH1, HNRNPF, LSM7, SUGP2, XRN2
GO:0007155∼cell adhesion	6.68E-05	4.1	ALCAM, APP, CCN1, CCN2, CDH2, CTNNAL1, CTNNB1, DSG2, IGFBP7, LAMB2, LGALS3BP, PXN
GO:0002576∼platelet degranulation	7.18E-05	9.9	APLP2, APP, LAMP2, LGALS3BP, QSOX1, SPARC, TGFB2
GO:0042060∼wound healing	2.12E-04	11.0	DCBLD2, EGFR, LOX, SPARC, TGFB2, TPM1
GO:0000398∼mRNA splicing, via spliceosome	7.99E-04	5.3	BUD31, HNRNPA2B1, HNRNPH1, HNRNPH2, HNRNPF, LSM7, SRRT, YBX1
GO:0006397∼mRNA processing	1.39E-03	5.7	CCAR2, HNRNPA2B1, HNRNPLL, QKI, SUGP2, XRN2, ZC3H14,
GO:0008285∼negative regulation of cell proliferation	1.51E-03	3.7	ADAMTS1, CTNNB1, CUL1, IGFBP7, ITGA1, NF2, PKP2, QSOX1, SOD2, TGFB2
GO:0030198∼extracellular matrix organization	2.20E-03	5.2	APP, CCN1, ERO1A, ITGA1, LAMB2, LOX, SPARC,
GO:0098911∼regulation of ventricular cardiac muscle cell action potential	2.41E-03	39.8	DLG1,DSG2, PKP2
GO:2001241∼positive regulation of extrinsic apoptotic signaling pathway in absence of ligand	3.94E-03	31.3	CTNNA1, DAPK3, TGFB2

*^*a*^Gene ontology enrichment analysis of host proteins of interest (Supplementary Table S8) using DAVID Bioinformatics Resources. ^*b*^Top 10 enriched gene ontology (GO) biologically processes (Modified Fisher’s exact test: *p* < 0.05). ^*c*^Gene names of differentially expressed host proteins categorized into the GO biological process indicated.*

**TABLE 3:** Gene ontology enrichment analysis of proteins differentially expressed at 3 h after VZV^a^ infection.

**GO term (biological process)^b^**	***p*-value**	**Fold enrichment**	**Host proteins^c^**
**Upregulated proteins**			
GO:0030198∼extracellular matrix organization	6,68E-04	8,42	COL4A1, COL4A2, COL5A2, GFAP, FBN1, HSPG2
GO:0070537∼histone H2A K63-linked deubiquitination	0,0106	183,51	BRCC3, USP16
GO:1904714∼regulation of chaperone-mediated autophagy	0,0177	110,11	EEF1A2, GFAP
GO:0051180∼vitamin transport	0,0177	110,11	GC, APOA1
GO:0038063∼collagen-activated tyrosine kinase receptor signaling pathway	0,0212	91,75	COL4A1, COL4A2
GO:0030574∼collagen catabolic process	0,0219	12,90	COL4A1, COL4A2, COL5A2
GO:0031102∼neuron projection regeneration	0,0282	68,81	GFAP. APOA1
GO:0016559∼peroxisome fission	0,0351	55,05	MFF, ACOT8
GO:0051301∼cell division	0,0363	3,93	BRCC3, ZFYVE19, NEK2, USP16, CDC23
**Downregulated proteins**			
GO:0010501∼RNA secondary structure unwinding	0,0182	95,40	DHX36, DDX19A

*^*a*^Gene ontology enrichment analysis of host proteins significantly differentially expressed (adjusted *p*-value < 0.05) using DAVID Bioinformatics Resources. ^*b*^Enriched gene ontology (GO) biologically processes (Modified Fisher’s exact test: *p* < 0.05). ^*c*^Gene names of differentially expressed host proteins categorized into the GO biological process indicated.*

### Comparison of the Host Proteome Between Productive HSV-1 and VZV Infection

**FIGURE 9:**
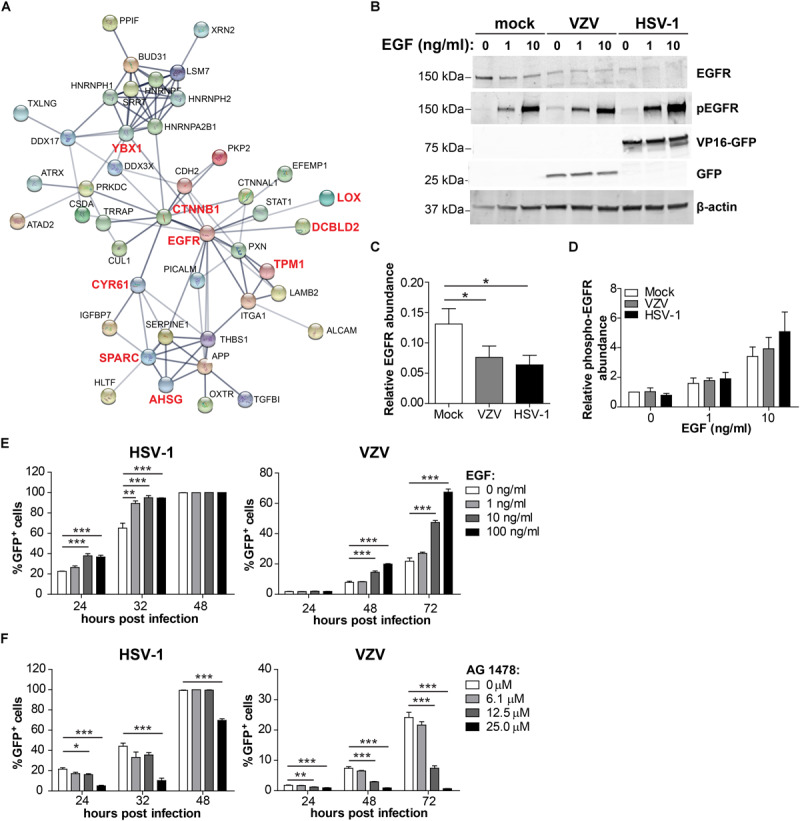
Comparison of the host proteome during productive HSV-1 and VZV infection of ARPE-19 cells. **(A)** Protein-protein interaction network (STRING Database) analysis of the conserved 7 affected host proteins and the significant differentially expressed host proteins in the same Gene Ontology biological process (Tables 1, 2). **(B)** ARPE-19 cells were infected with VZV.BAC-GFP or HSV-1.VP16-GFP for 24 h, stimulated with the indicated dose of EGF for 30 min and analyzed by western blotting using antibodies directed to EGFR, phosphorylated EGFR (p-EGFR), GFP and β-actin. **(C,D)** Signal intensity of EGFR, p-EGFR and β-actin was quantified and the average ratios EGFR/β-actin (C, *n* = 5 experiments) and p-EGFR/β-actin (D; *n* = 3 experiments) ± SEM are shown. **(E,F)** ARPE-19 cells were infected with cell-free HSV-1.VP16-GFP or cell-free VZV.BAC-GFP for 4 h, treated with EGF **(E)** or specific EGF inhibitor AG 1478 **(F)** and GFP expression was analyzed at indicated times after infection by flow cytometry. Two independent experiments performed, data (average ± SEM) shown from one representative experiment (*n* = 3 replicates). *p < 0.05, **p < 0.01, ***p < 0.001 by one-way ANOVA and Bonferroni’s multiple comparison correction.

To identify host factors and pathways that are critical for productive αHHV infection in ARPE-19 cells, we analyzed which host cell proteins and/or pathways were affected by both VZV and HSV-1. Because our VZV MS analysis (3,714 human proteins identified) was more sensitive compared to our HSV-1 MS analysis (1,526 host proteins identified), we cannot perform quantitative comparison between both datasets. However, qualitative comparisons, such as cellular pathways or host proteins affected by both viruses, are legitimate and informative. Although qualitative comparison is likely to underestimate the overlap in proteomic changes between both αHHVs, we observed shared effects on various host cell pathways (Tables 1–3). Additionally, the MS data obtained demonstrated that both viruses significantly affected the expression of 11 host proteins, including one upregulated and 10 downregulated proteins (Table 4). To investigate the function of these host proteins, we used the STRING database resource (Szklarczyk et al., 2017) to construct a protein-protein interaction network including all significant differentially expressed host proteins included within the same Gene Ontology biological processes. Notably, nine of 11 host proteins affected by both VZV and HSV-1 infection were involved in epidermal growth factor receptor (EGFR) signaling (Figure 9A).

**TABLE 4:** Host proteins affected by both HSV-1 and VZV infection.

**Gene Name**	**Protein description**	**Direction^a^**
AHSG	Alpha-2-HS-glycoprotein	Upregulated
CTNNB1	Catenin beta-1	Downregulated
CYR61	Cysteine-rich angiogenic inducer 61	Downregulated
DCBLD2	Discoidin, CUB and LCCL domain-containing protein 2	Downregulated
G3BP2	Ras GTPase-activating protein-binding protein 2	Downregulated
LOX	Protein-lysine 6-oxidase	Downregulated
SPARC	Secreted protein acidic and rich in cysteine (also known as osteonectin)	Downregulated
TPM1	Tropomyosin alpha-1 chain	Downregulated
TXNDC17	Thioredoxin domain-containing protein 17	Downregulated
YBX1	Y-box-binding protein 1	Downregulated
YBX3	Y-box-binding protein 3	Downregulated

*^*a*^Effect of HSV-1 and VZV infection on expression levels of indicated host protein.*

Given the discrepancy between the sensitivity of our HSV-1 and VZV MS analyses, we additionally compared our VZV MS dataset with a previously published HSV-1 MS dataset of similar depth (3,714 and 4,030 human proteins identified, respectively) (Kulej et al., 2017). Despite the different cell types used - human retinal pigment epithelial cells (VZV, this study) and human foreskin fibroblasts (HSV-1, Kulej et al., 2017) – we identified 56 proteins affected by both HSV-1 and VZV infection (Supplementary Figure S9A), including 12 upregulated proteins and 44 downregulated proteins. Additionally, both viruses affected similar cellular processes including RNA processing, extracellular matrix organization and cell adhesion (Supplementary Figure S9B) – comparable to the results obtained from the comparison of our HSV-1 and VZV MS datasets obtained from ARPE-19 cells (Tables 1, 2). Strikingly, also the network analysis of the 56 proteins affected by virus infection in our VZV MS data and the previously published HSV-1 MS data demonstrated that 36 of 56 proteins were involved in EGFR signaling (Supplementary Figure S9C). Thus, EGFR signaling appears to be a major pathway that is affected by both VZV and HSV-1 infection in multiple cell types.

VZV infection directly reduced EGFR expression (Supplementary Table S8) and HSV-1 was previously reported to downregulate EGFR expression (Shu et al., 2015; Kulej et al., 2017) (Supplementary Table S9). To confirm the MS data and to investigate the effect of VZV and HSV-1 infection on EGFR expression as well as EGF-induced EGFR phosphorylation, virus-infected ARPE-19 cells were treated with EGF and subsequently analyzed by WB. Green fluorescent protein (GFP)-expressing HSV-1 (HSV-1.VP16-GFP) and VZV (VZV.BAC-GFP) were used, both of which replicate similar to wild-type virus strains (La Boissiere et al., 2004; Zhang et al., 2010). EGF treatment induced degradation of EGFR in mock-infected ARPE-19 cells (Figure 9B), as reported earlier (Henriksen et al., 2013). Notably, both VZV and HSV-1 infection decreased EGFR expression, but did not impair EGF-induced EGFR phosphorylation (Tyr1068) (Figures 9C,D and Supplementary Figure S10). Thus, both αHHV maintained functional EGFR signaling in the presence of reduced EGFR expression.

To investigate whether stimulation of EGFR signaling could be pro-viral for both αHHV, ARPE-19 cells were infected with cell-free HSV-1.VP16-GFP or VZV.BAC-GFP and at 4 hpi cells were treated with or without EGF and infection frequencies determined at different hpi by flow cytometry. EGF treatment increased the frequency of both HSV-1-infected and VZV-infected cells in a dose-dependent manner in two independent experiments (Figure 9E and Supplementary Figure S11A). Next, we determined whether inhibition of basal EGFR signaling was anti-viral for both αHHV. For this, ARPE-19 cells were infected with cell-free HSV-1.VP16-GFP or VZV.BAC-GFP and at four hpi cells were treated with the specific EGFR inhibitor AG1478 and infection frequencies determined at different hpi by flow cytometry. No cytotoxicity was observed in cells treated with <50 μM AG1478 (Supplementary Figure S12). Indeed, inhibition of basal EGFR signaling severely reduced HSV-1 and VZV replication in ARPE-19 cells in a dose-dependent manner (Figure 9F and Supplementary Figure S11B). In conclusion, these data demonstrate the important role of EGFR in productive HSV-1 and VZV infection and replication in human epithelial cells.

## Discussion

This study provides a comprehensive overview of the temporal changes in virus and host protein expression during productive infection of human retinal pigment epithelial cells with the closely related αHHV HSV-1 and VZV. This is the first study that investigated the global pattern of changes in protein expression during productive VZV infection, quantifying 51 VZV proteins and 3,714 host proteins from 0 to 24 hpi at 3-h intervals. While other studies have analyzed proteomes of HSV-1 infected cells (Antrobus et al., 2009; Berard et al., 2015; Kulej et al., 2017), the current study investigated more samples at shorter time intervals post-infection and quantified 51 viral proteins and 1,526 host proteins from 0 to 12 hpi at 2-h intervals. Importantly, conservative comparison of host proteomes after HSV-1 and VZV infections revealed viral interference with similar cellular processes and identified a conserved role for EGFR signaling in both HSV-1 and VZV replication.

We quantified 70 and 74% of the canonical HSV-1 and VZV proteins. The inability to detect all canonical HSV-1 or VZV proteins was not dependent on protein size and predicted number of peptides obtained after trypsin digestion (Supplementary Figure S13), but may potentially be attributed to transcript expression levels of the corresponding viral proteins (Cohrs et al., 2003; Tombacz et al., 2017). Additionally, intrinsic protein characteristics like solubility during digestion procedure and the chemical properties of the obtained peptides after digestion, like hydrophobicity and ionization efficiency, may have impeded detection of all viral proteins in our experimental set-up (Lubec and Afjehi-Sadat, 2007). While our 70–74% coverage of HSV/VZV proteomes is comparable to that obtained for other viruses in previous studies (range: 61–81%) (Bell et al., 2013; Weekes et al., 2014; Berard et al., 2015; Ersing et al., 2017; Kulej et al., 2017; Soday et al., 2019), continuous development of more sensitive mass spectrometers is likely to increase viral protein coverage in future studies.

Temporal analysis of HSV-1 and VZV proteomes enabled examination of the expression patterns of viral proteins during productive infection of ARPE-19 cells, a well-described human retina pigmented epithelial cell line highly susceptible to both αHHV (Dunn et al., 1996; Ouwendijk et al., 2014). The kinetic class of HSV-1 genes is mainly defined based on mRNA expression profiling, often combined with specific inhibitors of protein synthesis or viral DNA replication to enrich for α gene mRNAs and differentiate between γ1 and γ2 genes (Roizman et al., 2013). Our proteomic analysis demonstrated that the pattern of HSV-1 protein expression largely corresponded to the kinetic class of their transcripts. Interestingly, while α gene products ICP0 and ICP4 are among the first viral proteins expressed in newly infected cells (Roizman et al., 2013), we and others (Lium and Silverstein, 1997) consistently detected both HSV-1 proteins only at 4–6 hpi by MS and WB. In addition to assay sensitivity and protein abundance, a recent study suggests that these observations could also be caused by high cell-to-cell variability in susceptibility to HSV-1 infection, even in a monoculture (Drayman et al., 2019). Indeed, flow cytometric analysis of 6 HSV-1 proteins indicated that not all virus-infected ARPE-19 cells expressed the analyzed viral proteins at the same time and to the same abundance (Supplementary Figure S14), indicating a need for future studies using single-cell mass-spectrometric analyses (Budnik et al., 2018).

The pattern of VZV protein expression did not conclusively demonstrate temporal expression of viral proteins, with most VZV proteins only significantly expressed and measured by MS relatively late during infection (>9 hpi). By contrast, a previous study detected VZV ORFs 23, 29, 61, 62, 63, and 68 (gE) at earlier times compared to our analysis, and showed that newly produced infectious virus is released by 12 hpi (Reichelt et al., 2009). Most likely, these discrepancies are caused by differences in inoculum format, in addition to differences in assay sensitivity. The use of cell-associated virus by Reichelt et al. (2009) would expose cells to a continuous and much higher virus dose than the multiplicity of infection of one used in this study. Alternatively, the high amount of non-infectious relative to infectious VZV particles (Carpenter et al., 2009) in the cell-free virus inoculum may result in asynchronous infection due to competition for receptor binding. Therefore, application of recently developed methods that enable simultaneous analysis of virus/host transcriptomes and proteomes at the single cell level are required to provide a definite kinetic classification of VZV genes and corresponding proteins (Budnik et al., 2018).

Both HSV-1 and VZV infection affected cellular transcription and RNA processing, cell division and proliferation, and the ECM, suggesting that manipulation of these processes is critical for productive αHHV infection. These findings are consistent with previously reported pathways affected by HSV-1 infection in human foreskin fibroblasts (HFF) and embryonic kidney epithelial (HEK293) cells (Berard et al., 2015; Kulej et al., 2017); although others reported more pronounced effects of HSV-1 on immune responses and cell death (Kulej et al., 2017). In our study, ECM remodeling was most severely affected by both VZV and HSV-1 infection, consistent with previously reported ECM remodeling by HSV-1 (Lashgari et al., 1987; Visser et al., 1989). Possibly, αHHV-mediated remodeling of the ECM promotes cell-to-cell spread or release of virus at the cell surface. This is supported by a recent study showing that HSV-1 infection induced heparanase expression to increase release of infectious virus into the supernatant (Hadigal et al., 2015). Further, VZV infection downregulated host proteins involved in cell adhesion (Table 2), which could stimulate cell-to-cell spread of this highly cell-associated virus.

ECM remodeling by HSV-1 and VZV infection affects cellular signal transduction pathways. ECM components bind secreted soluble factors, like growth factors and cytokines, and interact with cell surface receptors (Brekken and Sage, 2000). Herein, we showed that both viruses reduced expression of EGFR, discoidin CUB and LCCL domain-containing protein 2 (DCBLD2), LOX and SPARC. EGFR is the prototypic receptor tyrosine kinase (RTK), comprising a family of cell surface receptors involved in growth factor, cytokine and hormone signaling (Chen et al., 2018). DCBLD2 interacts with multiple RTKs, including EGFR, insulin receptor, vascular endothelial growth factor (VEGF) receptor and platelet-derived growth factor (PDGF) receptor, to regulate their activity (Li et al., 2016). Moreover, DCBLD2 is phosphorylated by activated EGFR to recruit TRAF6 and activate AKT (Feng et al., 2014). SPARC binds to and regulates activity of growth factors such as PDGF, VEGF and fibroblast growth factor two (Brekken and Sage, 2000). At later stages of infection, VZV downregulated various other types of cell surface receptors, including the oxytocin receptor (OXTR), GDNF family receptor alpha-1 (GFRA1) and the poliovirus receptor (PVR; CD155), suggesting a more broad effect of virus infection on cell surface receptors. Assembly of mature herpesvirus virions, which involves clathrin- and caveola-dependent endocytosis of viral glycoproteins from the plasma membrane (Albecka et al., 2016) could potentially result in non-specific loss of receptors from the cell surface of HSV-1- and VZV-infected cells. Future studies are warranted to determine the specificity and function of ECM remodeling and its associated pleiotropic effects on signal transduction pathways during productive αHHV infection.

We demonstrated that EGFR signaling is important for efficient HSV-1 and VZV replication in ARPE-19 cells. EGFR is a member of the ErbB RTK family, expressed by a wide variety of cells and involved in regulation of fundamental cellular processes including proliferation, migration, cell death and innate immune signaling (Yamashita et al., 2012; Lupberger et al., 2013; Ho et al., 2017). Consequently, a plethora of viruses modulate EGFR expression and/or signaling to promote viral entry into the host cell, induce host cell survival and proliferation or modulate antiviral immune responses (Ho et al., 2017). Among human herpesviruses, Kaposi’s sarcoma-associated herpesvirus and Epstein-Barr virus upregulate and activate EGFR to promote cell proliferation (Kung et al., 2011; Cheng et al., 2015). Human cytomegalovirus (HCMV) utilizes EGFR as a co-receptor for cell entry, and specifically regulates surface expression of EGFR to promote productive or latent infections (Wang et al., 2003; Buehler et al., 2016). Herein, we showed that while both HSV-1 and VZV downregulated total cellular EGFR expression levels, this did not impair EGF-induced EGFR phosphorylation. Our data are consistent with previously reported downregulation of total and cell surface EGFR in HSV-1-infected cells (Shu et al., 2015), but suggest that HSV-1 and VZV retain sufficient functional EGFR on the cell surface to support EGFR signaling in infected cells. Similar to HCMV, HSV-1 (and possibly VZV) may use EGFR signaling-mediated remodeling of the actin cytoskeleton to enhance virus uptake in host cells (Zheng et al., 2014). However, our data suggest that EGF also promotes HSV-1 and VZV replication at post-entry stages. The mechanism by which EGFR signaling promotes HSV-1 and VZV replication during productive infection, and the role of EGFR signaling in neuronal latency will be intriguing avenues to follow-up in future studies.

The limitations of this study are the sensitivity of MS analysis and inevitable required modifications in the experimental set-up for VZV compared to HSV-1. Newly developed more sensitive MS and/or combined single-cell transcriptomics and proteomics are required to obtain better proteome coverage and account for intercellular variability in αHHV infection (Budnik et al., 2018). Furthermore, the VZV proteomic analysis quantified more host proteins (3,714) compared to HSV-1 (1,526). While normalized expression levels of host proteins detected in both datasets correlated significantly (Supplementary Figure S15A), greater log_2_-fold changes in protein expression were found in the VZV dataset (Supplementary Figures S15B,C) indicating higher sensitivity. Consequently, quantified protein abundances between both analyses are not directly comparable and prevent quantitative comparisons between viral and host proteomes. Reduced sensitivity of the HSV-1 proteomic analysis most likely resulted in an underestimated effect of virus infection on the host cell proteome. However, combined hierarchical cluster analysis and statistical analysis of differentially expressed host proteins within each viral database – which is dependent on variation, but not dynamic range (log_2_-fold change) of data – enabled identification of cellular processes affected by both HSV-1 and VZV. Despite these limitations we were able to identify 11 host proteins that were significantly affected by both viruses and are therefore presumed to be conserved essential host factors for αHHV replication in ARPE-19 cells. Moreover, we identified substantial overlap between host proteins and cellular pathways impacted by VZV and HSV-1 infection when our VZV dataset was compared to a previously published HSV-1 dataset with similar sensitivity (Supplementary Figure S9), despite differences in cell type used between both studies.

In conclusion, our data revealed the temporal expression pattern of VZV and HSV-1 proteins during productive infection in human retinal pigment epithelial cells. Comparative analyses of host proteomes during HSV-1 and VZV infection demonstrated that both viruses interfered with similar cellular processes, including ECM remodeling and RNA processing. Moreover, we demonstrate the important role for EGFR signaling in promoting productive HSV-1 and VZV infection. Overall, this study provides a temporal proteomic map of virus and host factors expressed during productive infection of HSV-1 and VZV and serves as a valuable resource for future studies aimed to identify key factors as potential target(s) for novel intervention strategies.

## Data Availability Statement

All data analyzed for this study are included in the article/Supplementary Material.

## Author Contributions

WO, LD, TL, and GV conceptualized the study. WO, LD, and H-JH, TL, and GV contributed to methodology. WO, LD, and H-JH provided the formal analysis and visualization. WO, LD, H-JH, and EH carried out the investigation. TR, SJ, and JH were responsible for the resources. WO  and GV wrote the manuscript.

## Conflict of Interest

H-JH was employed by Enpicom B.V.

The remaining authors declare that the research was conducted in the absence of any commercial or financial relationships that could be construed as a potential conflict of interest.

## Footnotes

1 https://software.broadinstitute.org/morpheus
