# Supplementary material for: Analysis of Virus and Host Proteomes During Productive HSV-1 and VZV Infection in Human Epithelial Cells
Source: Front Microbiol. 2020 May 29;11:1179. doi: 10.3389/fmicb.2020.01179 (PMC7273502; doi:10.3389/fmicb.2020.01179)
Supplement: Supplementary file 2 [file Table_2.DOCX]

| **S2 Table. Phosphorylation, methylation and sulfonation sites in HSV-1 proteins^a^** | | | |
| --- | --- | --- | --- |
| **Protein** | **Phosphorylated residue** | **Methylated residue** | **Sulfonated residue** |
| RL2 (ICP0) | S508 |  |  |
| RS1 (ICP4) | S80, S106, S157, S536 | D693 |  |
| UL10 | S391, S417 | D448. D452 |  |
| UL12 | S121, S604, S618 |  | S618 |
| UL13 |  | D164 |  |
| UL30 |  | E1221. E1222 |  |
| UL34 | S198 |  |  |
| UL36 |  | D1696 |  |
| UL39 | T308 |  |  |
| UL42 | S21, S328, S349, S468 | E26, E27 | S349, S352 |
| UL46 | S557 |  |  |
| UL49 | S71, S72 |  | S71 |
| UL56 | S101 |  |  |
| US1 | S22, S368, T374, S375, S376 |  | S363 |
| ^a^ Phosphorylated, methylated and sulfonated residues in HSV-1 proteins detected at 12 hour post-infection were determined by mass-spectrometry. | | | |
