# Supplementary material for: Analysis of Virus and Host Proteomes During Productive HSV-1 and VZV Infection in Human Epithelial Cells
Source: Front Microbiol. 2020 May 29;11:1179. doi: 10.3389/fmicb.2020.01179 (PMC7273502; doi:10.3389/fmicb.2020.01179)
Supplement: Supplementary file 4 [file Table_4.DOCX]

| **S4 Table. Phosphorylation, methylation and sulfonation sites in VZV proteins^a^** | | | |
| --- | --- | --- | --- |
| **Protein** | **Phosphorylated residue** | **Methylated residue** | **Sulfonated residue** |
| ORF4 | T146 | D230 |  |
| ORF8 | T33 | E37, E41, D44, D51 |  |
| ORF9 | S82, S118 |  |  |
| ORF12 | T546 |  |  |
| ORF27 | S35 |  |  |
| ORF33 | S484 |  |  |
| ORF49 | S34, T47 | E21. E28, E35, E38, E48, D49, D51 | S53, T54 |
| ORF62 | S16, S340, S358, S376, T381, S637, S649, S1290 | E337, D338, E343 | S379 |
| ^a^ Phosphorylated, methylated and sulfonated residues in VZV proteins detected at 24 hour post-infection were determined by mass-spectrometry. | | | |
