# Supplementary material for: Analysis of Virus and Host Proteomes During Productive HSV-1 and VZV Infection in Human Epithelial Cells
Source: Front Microbiol. 2020 May 29;11:1179. doi: 10.3389/fmicb.2020.01179 (PMC7273502; doi:10.3389/fmicb.2020.01179)
Supplement: Supplementary file 10 [file Data_Sheet_1.PDF]

Supplementary Material

1 Supplementary Figures

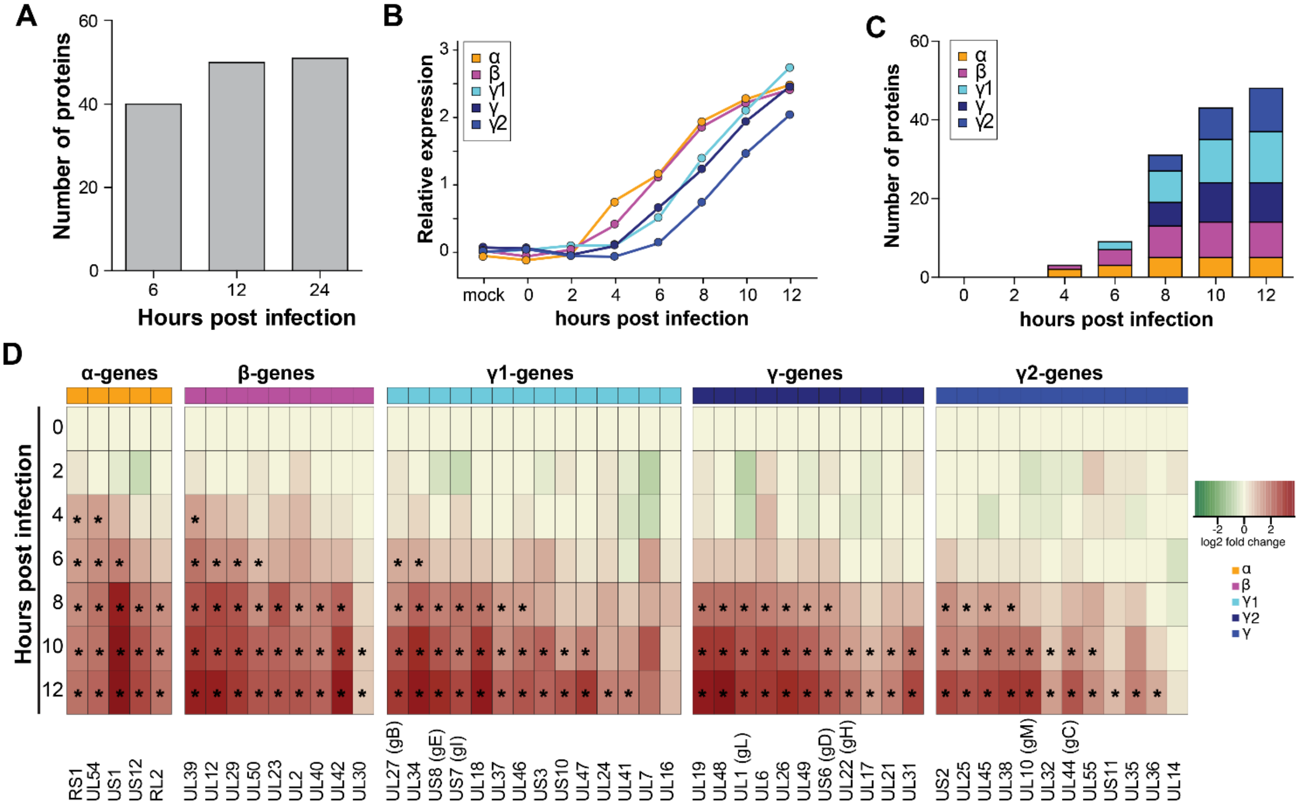

Supplementary Figure 1. Temporal analysis of the HSV-1 proteome during productive infection of ARPE-19 cells by mass spectrometry. HSV-1-infected ARPE-19 cells (F-strain, MOI=1) were analyzed by mass spectrometry. (**A**) Number of HSV-1 proteins detected in a single pilot experiment are indicated. (**B-D**) Three independent experiments were performed. (**B**) Relative protein expression (median log2-fold change) of α, β and γ (undefined, γ1 or γ2) genes. (**C**) Number of HSV-1 proteins expressed significantly more than baseline (mock-infected cells, adjusted p-value <0.05) per kinetic class. (**D**) Heatmap showing average log2-fold change in HSV-1 protein expression. Asterisks indicate significantly different protein abundance from baseline (mock-infected cells, adjusted p-value < 0.05). Reported kinetic classes of HSV-1 proteins are indicated.

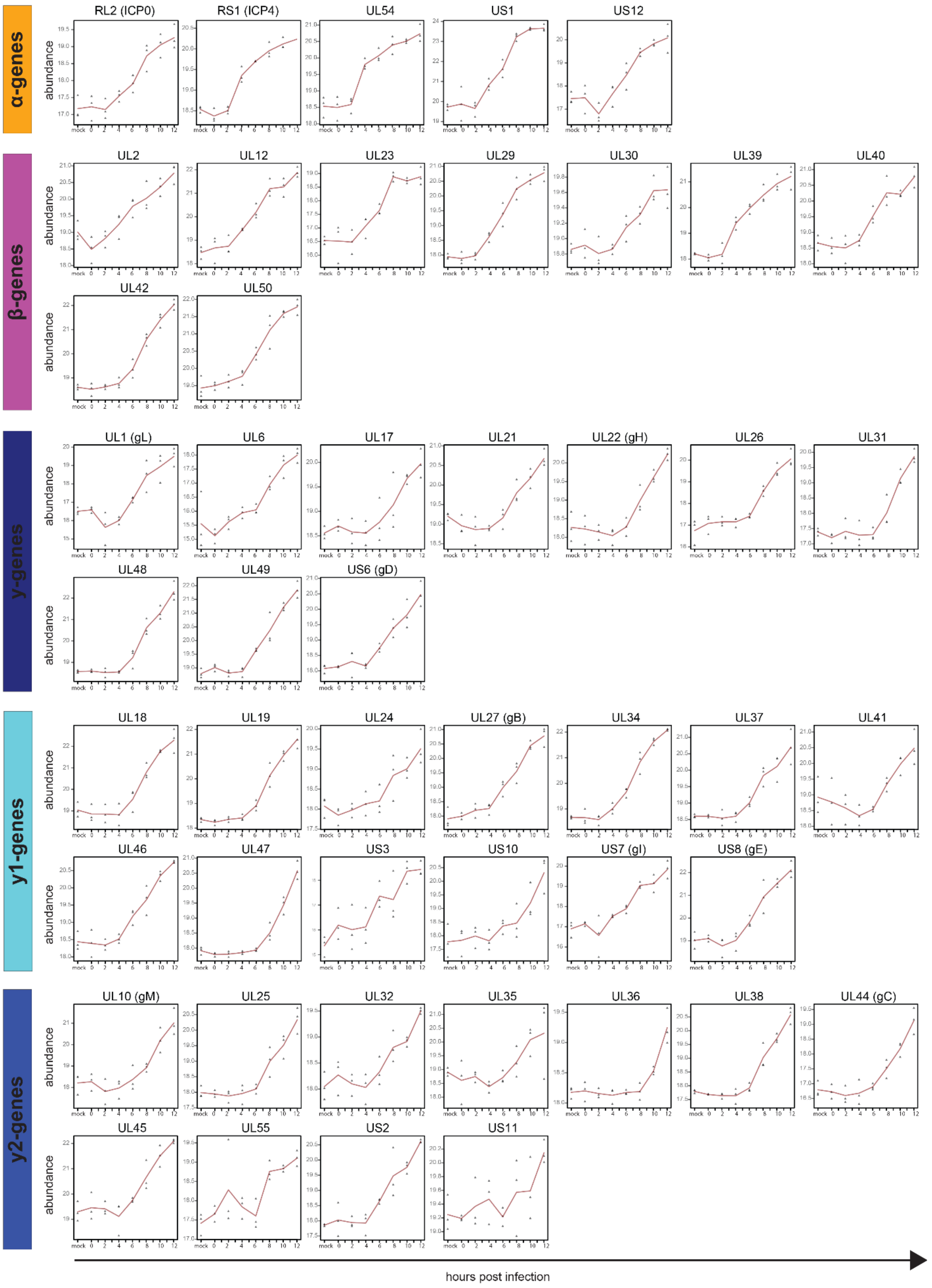

α-genes
RL2 (ICP0)
RS1 (ICP4)
UL54
US1
US12
β-genes
UL2
UL12
UL23
UL29
UL30
UL39
UL40
UL42
UL50
γ-genes
UL1 (gL)
UL6
UL17
UL21
UL22 (gH)
UL26
UL31
UL48
UL49
US6 (gD)
γ1-genes
UL18
UL19
UL24
UL27 (gB)
UL34
UL37
UL41
UL46
UL47
US3
US10
US7 (gI)
US8 (gE)
γ2-genes
UL10 (gM)
UL25
UL32
UL35
UL36
UL38
UL44 (gC)
UL45
UL55
US2
US11
abundance
mock 0 2 4 6 8 10 12
hours post infection

Supplementary Figure 2. Kinetics of HSV-1 protein expression during productive infection of ARPE-19 cells. Log2-transformed protein expression level (abundance) of individual α, β and γ (undefined, γ1 or γ2) HSV-1 gene products during productive infection of ARPE-19 cells, as determined by MS (n = 3 independent experiments). Grey triangles and red line indicate individual data points and average values.

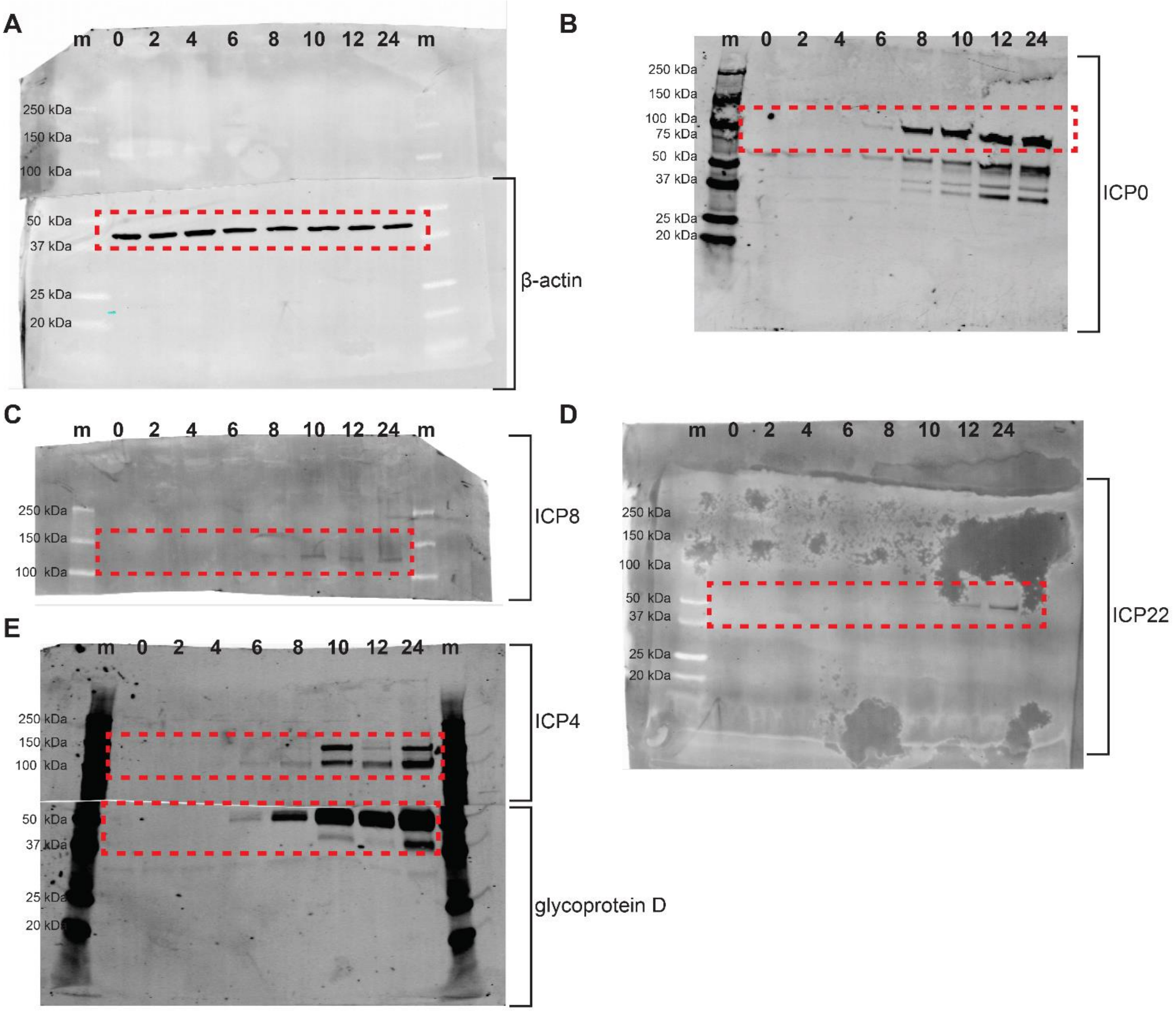

Supplementary Figure 3. Uncropped western blots for Figure 2. HSV-1-infected ARPE-19 cells (F-strain, MOI=1) were analyzed by western blotting using antibodies directed to β-actin (A), RL2 (ICP0; B), UL29 (ICP8; C), US1 (ICP22; D), RS1 (ICP4; E) and US6 (glycoprotein D; E). Dashed red boxes: areas shown in Figure 2. m, marker; 0 – 24, time (hrs) after infection.

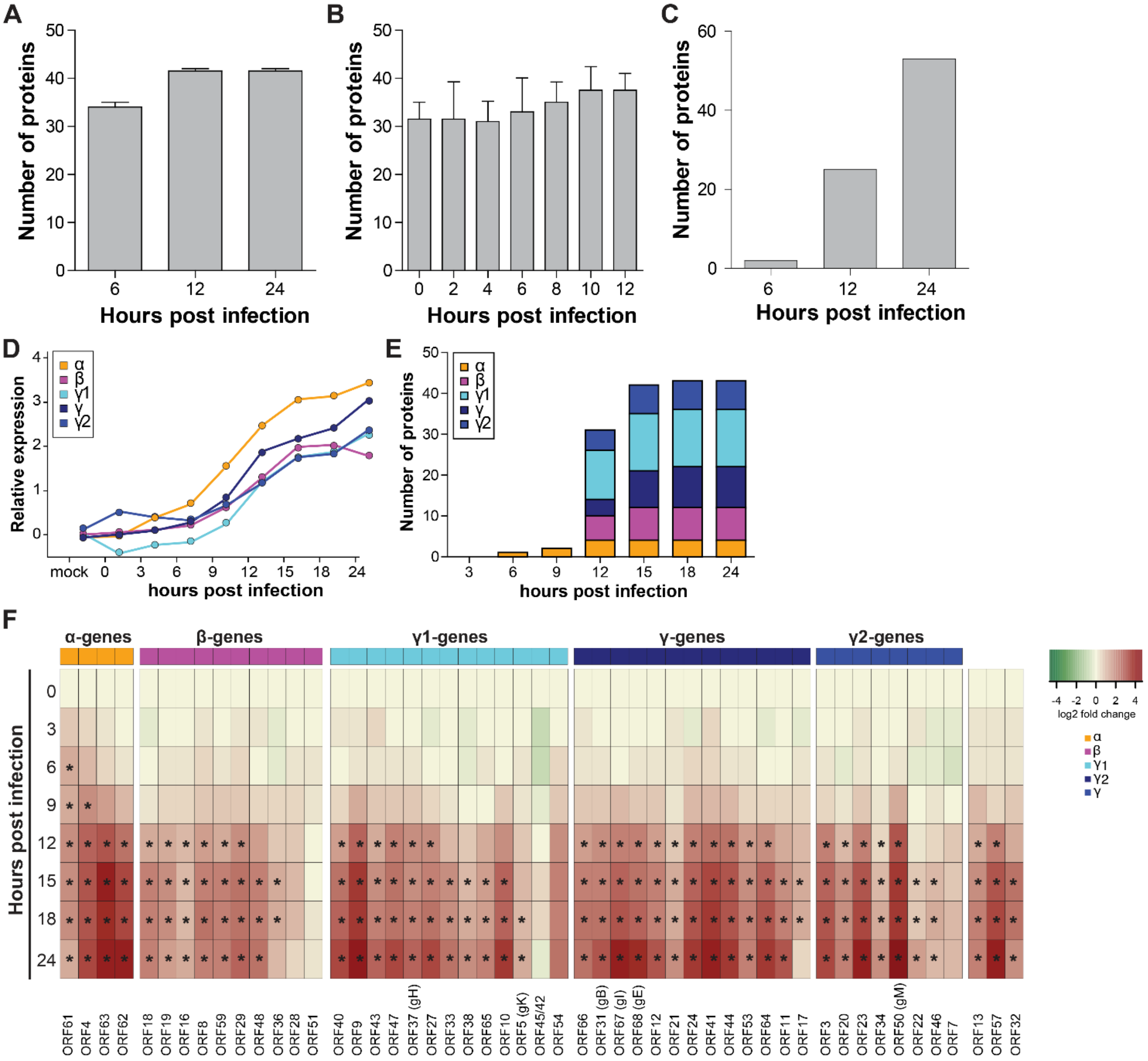

Supplementary Figure 4. Temporal analysis of the VZV proteome during productive infection of ARPE-19 cells by mass spectrometry. **(A-B)** Mass-spectrometric analysis of VZV proteins at 6, 12 and 24 hpi **(B)** or 0, 2, 4, 6, 8, 10 and 12 hpi **(C)**. Two independent experiments were performed. **(C-F)** $^{13}C_6$-L-Lysine and $^{13}C_6$-L-Arginine labelled ARPE-19 cells were infected with cell-free VZV (strain EMC-1, MOI=1), in the presence of $^{13}C_6$-L-Lysine and $^{13}C_6$-L-Arginine to label newly synthesized proteins, and analyzed by mass spectrometry. **(C)** Number of VZV proteins detected in a single pilot experiment. (**D-F**) Three independent experiments were performed. (**D**) Relative protein expression (median log2-fold change) of presumed α, β, and γ (undefined, γ1 or γ2) genes (solid lines). (**E**) Number of VZV proteins expressed significantly more than baseline (mock-infected cells, adjusted p-value <0.05) per kinetic class. (**F**) Heatmap showing log2-fold change in VZV protein abundance. Asterisks indicate significantly different protein expression from baseline (mock-infected cells, adjusted p-value < 0.05). Putative kinetic classes of VZV proteins, based on the kinetic class of their HSV-1 homologues, are indicated.

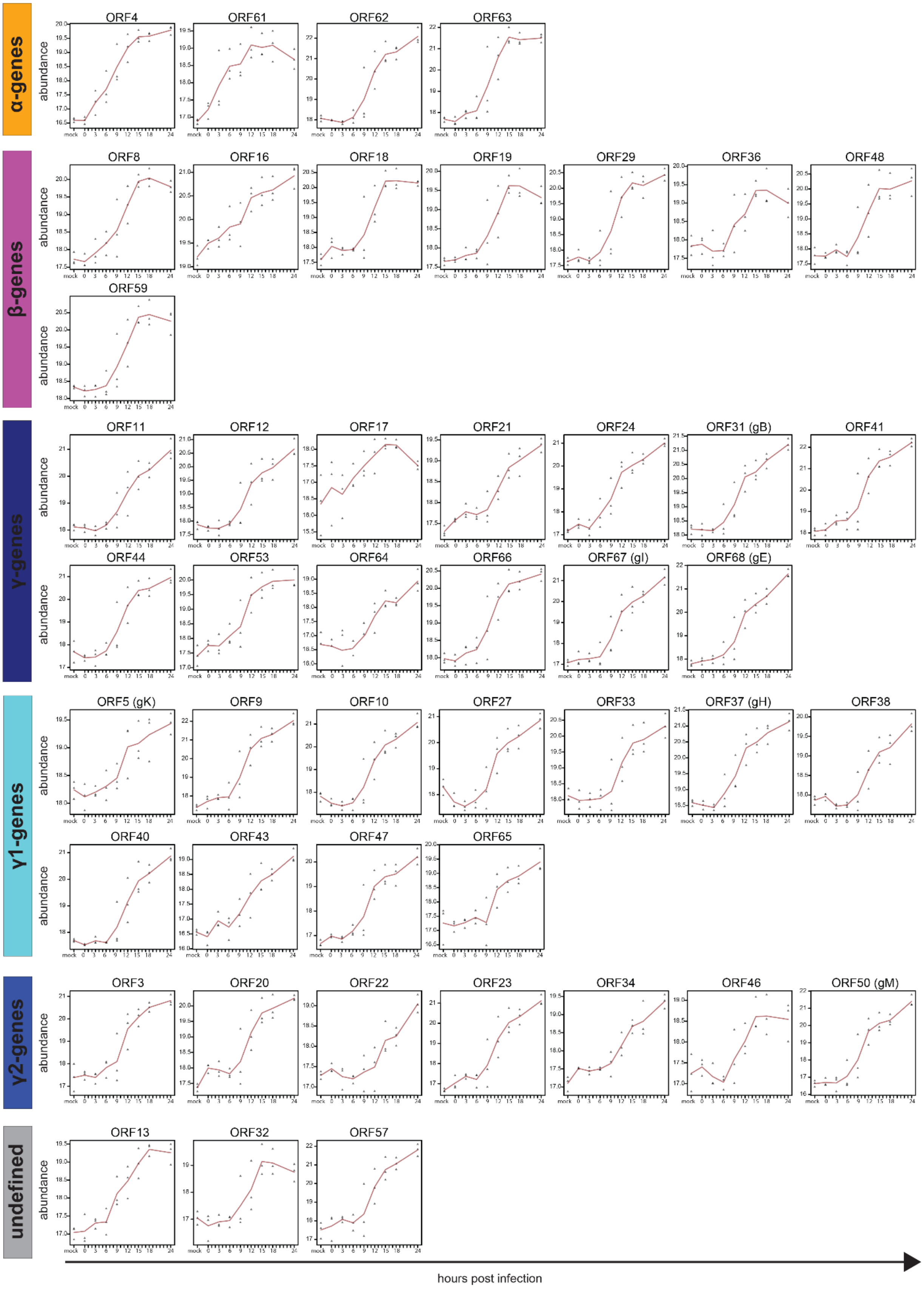
α-genes
abundance
ORF4
ORF61
ORF62
ORF63
β-genes
ORF8
ORF16
ORF18
ORF19
ORF29
ORF36
ORF48
ORF59
γ-genes
ORF11
ORF12
ORF17
ORF21
ORF24
ORF31 (gB)
ORF41
ORF44
ORF53
ORF64
ORF66
ORF67 (gI)
ORF68 (gE)
γ1-genes
ORF5 (gK)
ORF9
ORF10
ORF27
ORF33
ORF37 (gH)
ORF38
ORF40
ORF43
ORF47
ORF65
γ2-genes
ORF3
ORF20
ORF22
ORF23
ORF34
ORF46
ORF50 (gM)
undefined
ORF13
ORF32
ORF57
mock
hours post infection

Supplementary Figure 5. Kinetics of VZV protein expression during productive infection of ARPE-19 cells. Log2-trasnformedprotein expression levels (abundance) of individual putative α, β, γ (undefined, γ1 or γ2) VZV genes during productive infection of ARPE-19 cells, as determined by MS (n = 3 independent experiments). Grey triangles and red line indicate individual data points and average values.

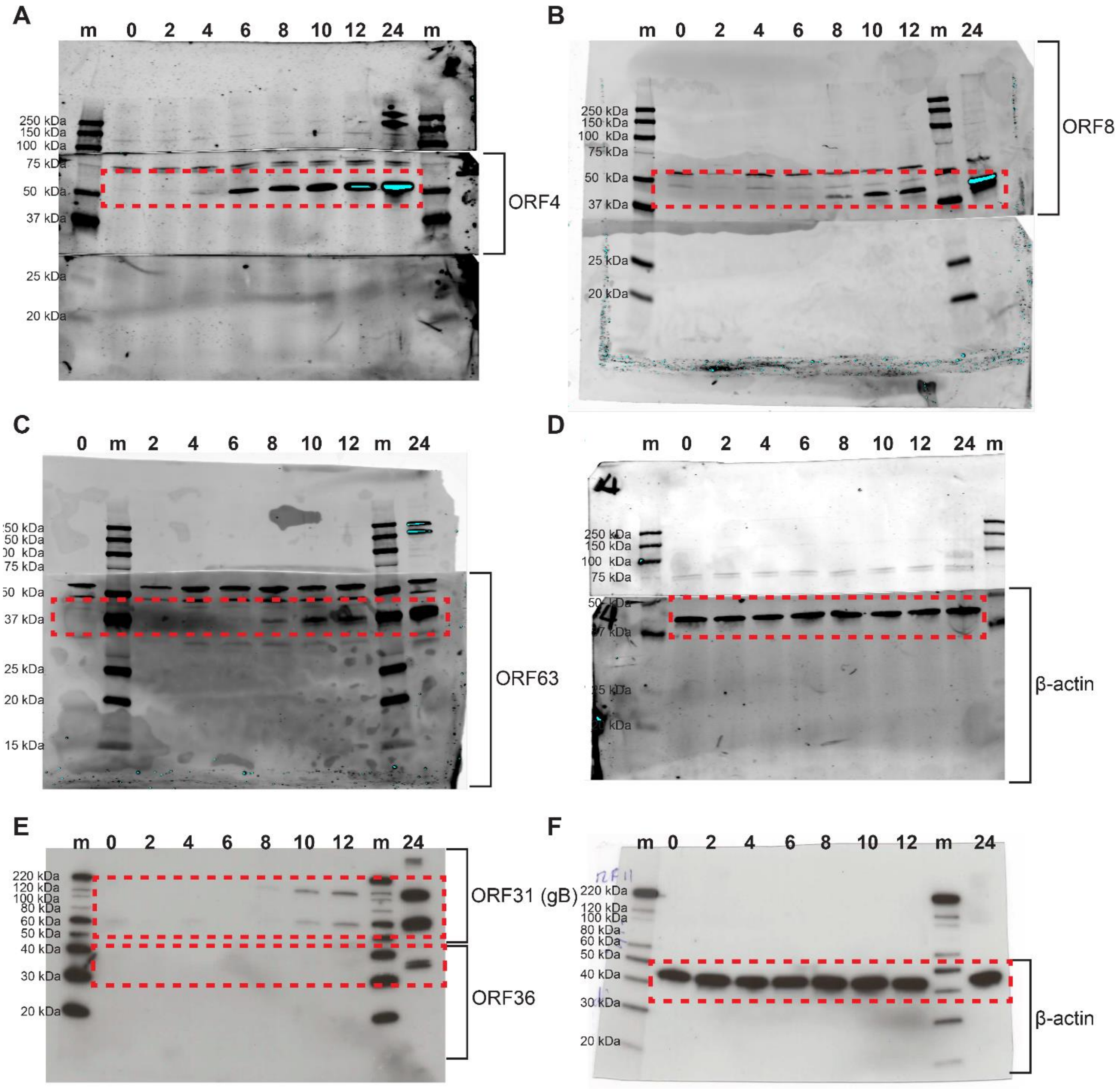

Supplementary Figure 6. Uncropped western blots for Figure 4. VZV-infected ARPE-19 cells (EMC-1 strain, MOI=1) were analyzed by western blotting using antibodies directed to ORF4 **(A)**, ORF8 **(B)**, ORF63 **(C)** and β-actin **(D)** using fluorescence signal visualization or stained for ORF31 and ORF36 **(E)** and β-actin **(F)** using chemiluminescent signal detection. Dashed red boxes: areas shown in Figure 4. m, marker; 0 – 24, time (hrs) after infection.

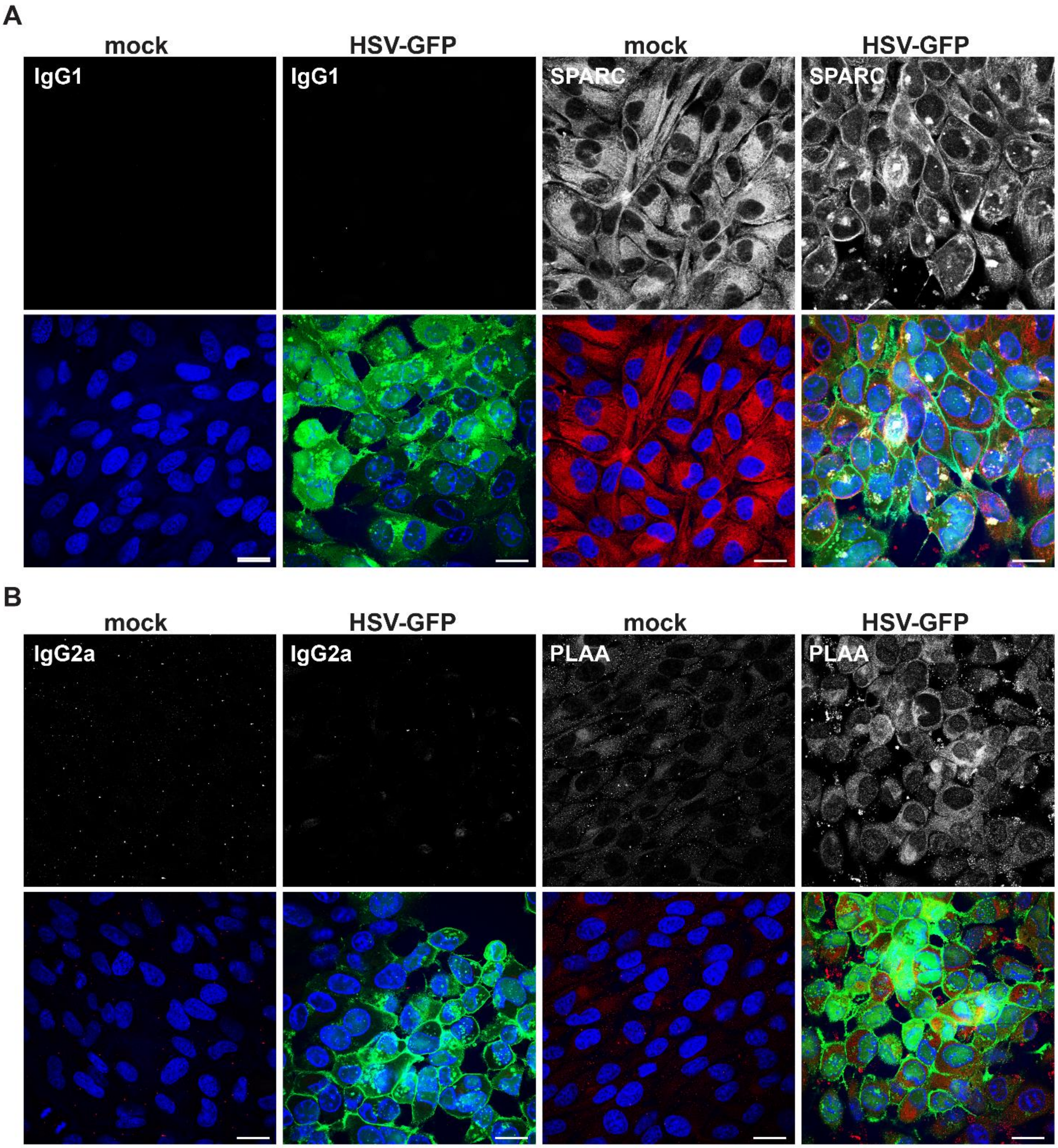

Supplementary Figure 7. Analysis of SPARC and PLAA expression in HSV-1 infected ARPE-19 cells. Confocal microscopy images of mock- and HSV-1-GFP-infected (green) cells stained for SPARC (red) **(A)**, PLAA (red) **(B)** and appropriate isotype control antibodies. Nuclei were stained with Hoechst (blue). Representative images are shown for n = 3 independent experiments. Scale bar: 20 µm.

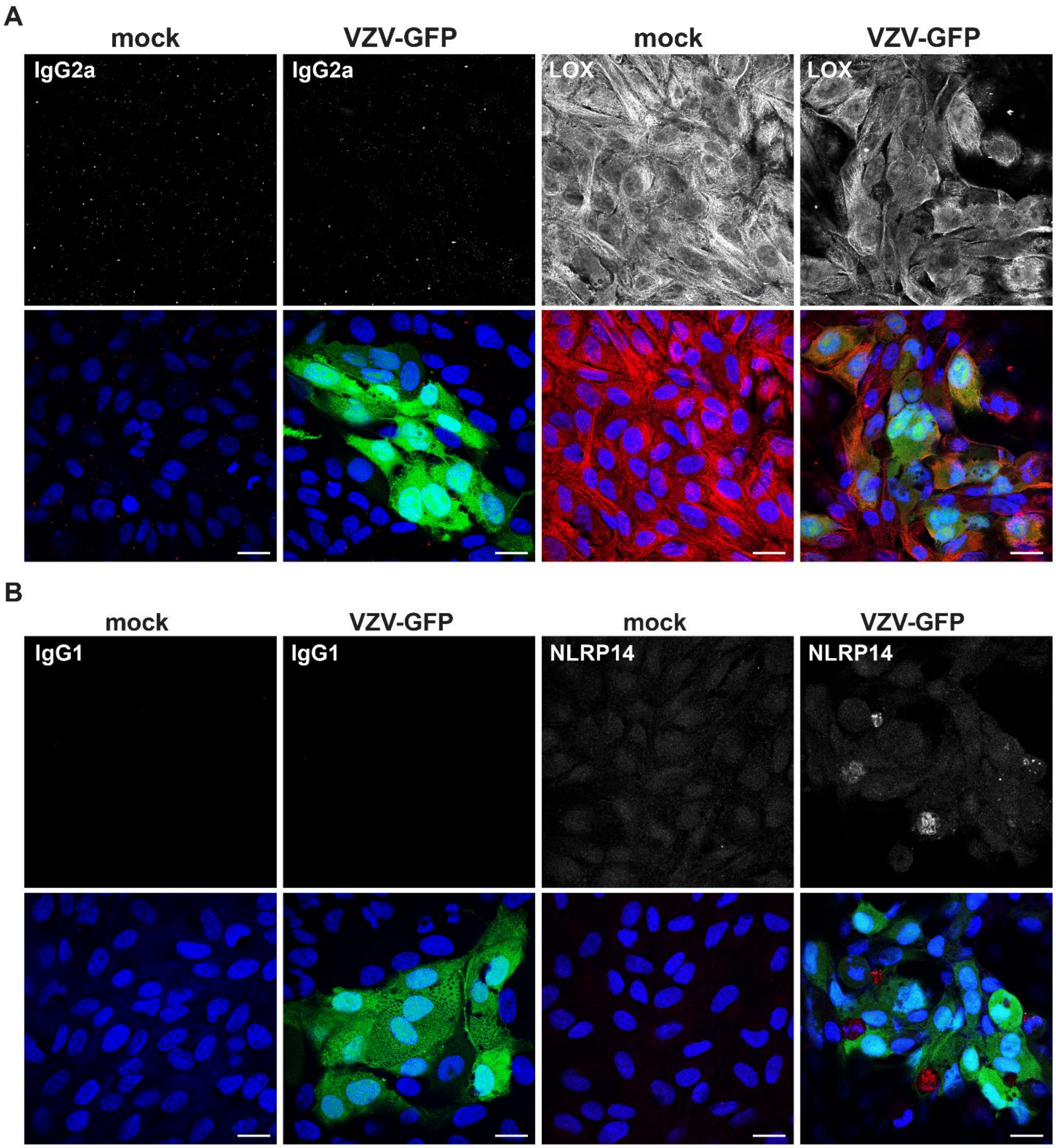

Supplementary Figure 8. Analysis of LOX and NLRP14 expression in VZV infected ARPE-19 cells. Confocal microscopy pictures of mock- and cell-free VZV.BAC-GFP (VZV-GFP; green) infected ARPE-19 cells stained for LOX (red) **(A)** and NLRP14 (red) **(B)** and appropriate isotype control antibodies. Nuclei were stained with Hoechst (blue). Representative images are shown for n = 3 independent experiments. Scale bar: 20 µm.

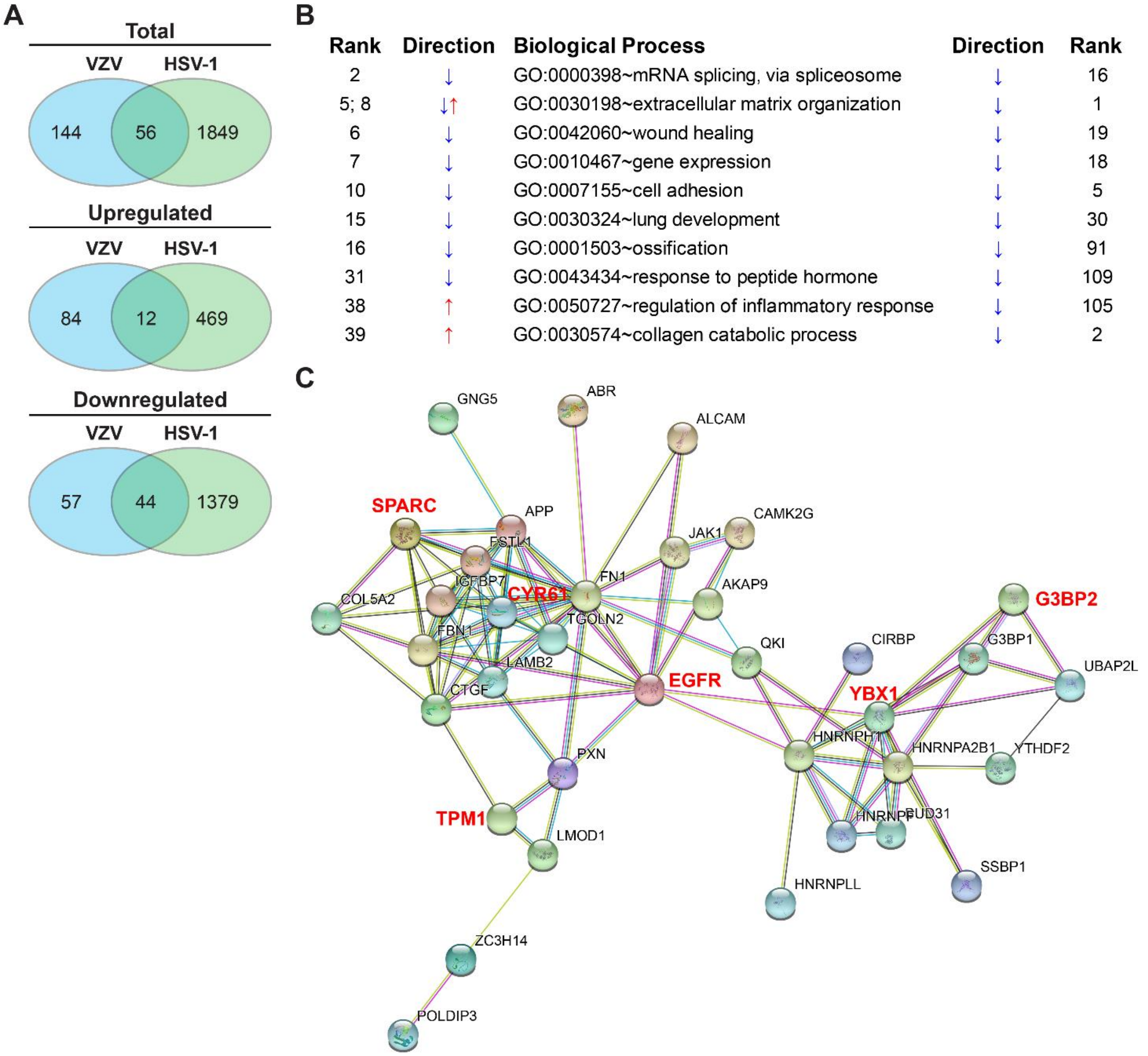

Rank	Direction	Biological Process	Direction	Rank
2	↓	GO:0000398~mRNA splicing, via spliceosome	↓	16
5; 8	↓↑	GO:0030198~extracellular matrix organization	↓	1
6	↓	GO:0042060~wound healing	↓	19
7	↓	GO:0010467~gene expression	↓	18
10	↓	GO:0007155~cell adhesion	↓	5
15	↓	GO:0030324~lung development	↓	30
16	↓	GO:0001503~ossification	↓	91
31	↓	GO:0043434~response to peptide hormone	↓	109
38	↑	GO:0050727~regulation of inflammatory response	↓	105
39	↑	GO:0030574~collagen catabolic process	↓	2

Supplementary Figure 9. Comparison of our VZV MS data and previously published HSV-1 MS data. Differentially expressed host proteins (adjusted p-value < 0.05) and host proteins clustering with virus proteins in VZV-infected ARPE-19 cells (current study) were compared with significantly differentially expressed host proteins (p<0.05) in HSV-1-infected HFF cells (Kulej et al., 2017). (**A**) Venn diagrams showing the overlap in host proteins affected by VZV and HSV-1 infections. (**B**) Comparison of biological processes affected by VZV and HSV-1 infection using DAVID functional annotation tool. (**C**) Protein-protein interaction network (STRING Database) analysis of the 56 host proteins affected by both VZV and HSV-1 infections.

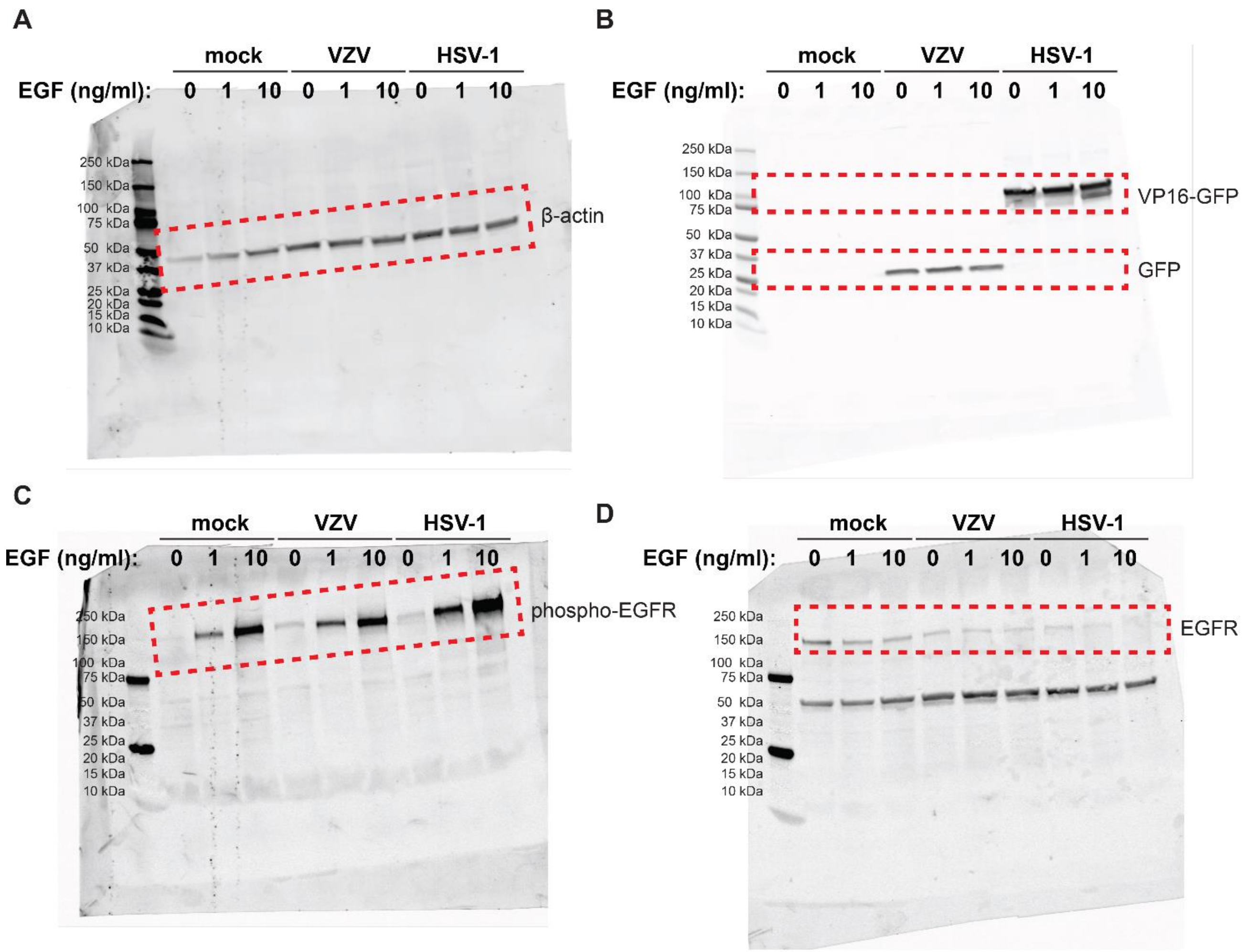

Supplementary Figure 10. Uncropped western blots for Figure 9B. ARPE-19 cells were infected with VZV.BAC-GFP or HSV-1.VP16-GFP for 24 hours, stimulated with the indicated dose of EGF for 30 minutes and analyzed by western blotting using antibodies directed to EGFR, phosphorylated EGFR (p-EGFR), GFP and β-actin. Dashed red boxes: areas shown in Figure 9B.

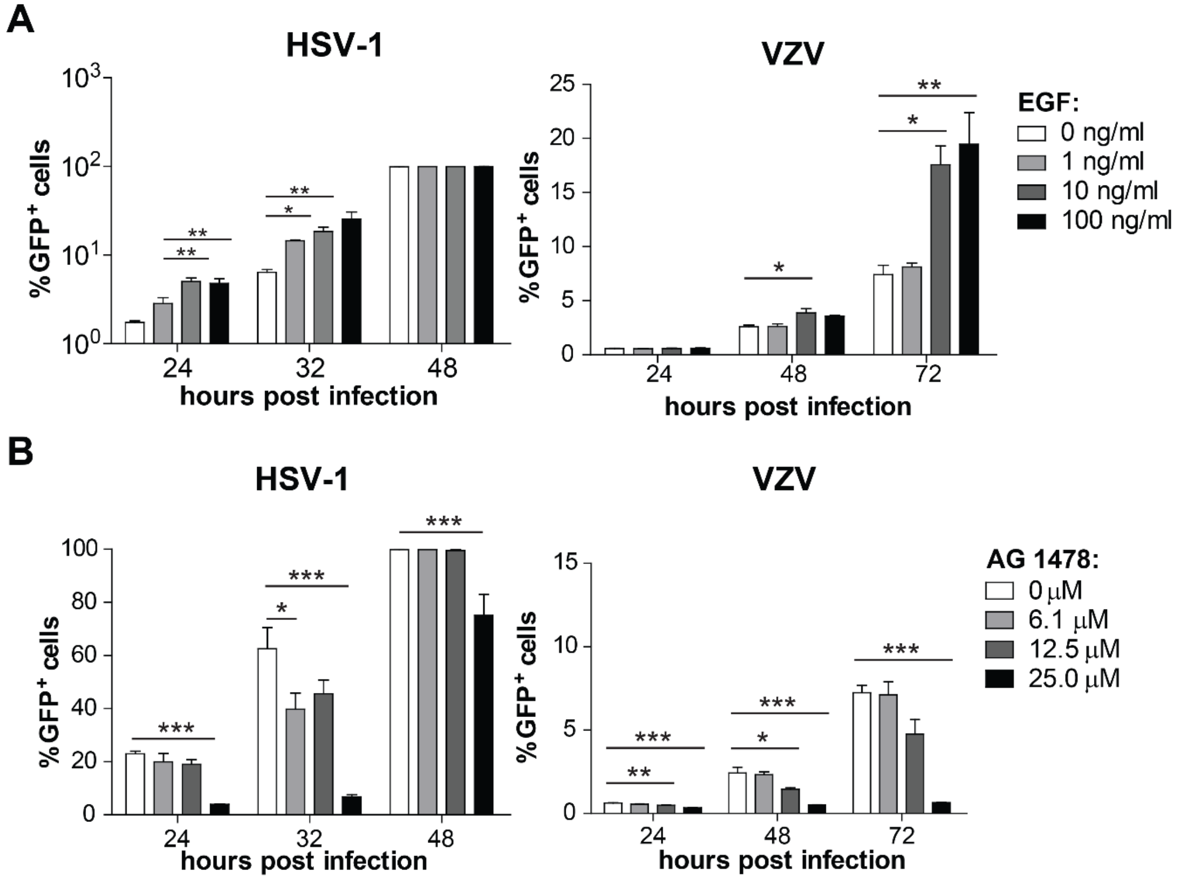

Supplementary Figure 11. Effect of EGFR stimulation and inhibition on HSV-1 and VZV replication. ARPE-19 cells were infected with cell-free HSV-1.VP16-GFP or cell-free VZV.BAC-GFP for 4 hours, treated with EGF **(A)** or specific EGF inhibitor AG1478 **(B)** and GFP expression was analyzed at indicated times after infection by flow cytometry. Data (average ± SEM) shown from an experiment (n=3 replicates) performed independently from the results shown in Figure 9. * p<0.05, **p<0.01, ***p<0.001 by one-way ANOVA and Bonferroni's multiple comparison correction.

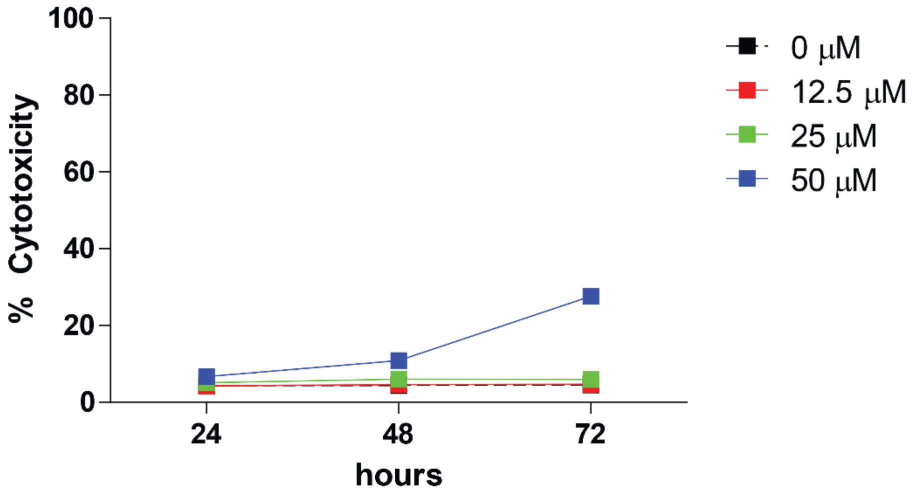

Supplementary Figure 12. Cytotoxicity profile of AG1478 in ARPE-19 cells. ARPE-19 cells were treated with indicated concentrations of EGFR inhibitor AG1478, supernatants were collected after 24, 48 and 72 hours and cell viability was analyzed by measuring lactate dehydrogenase (LDH) levels in supernatant. % Cytotoxicity = [LDH release AG1478-treated cells (OD @ 490 nm – 620 nm)] / [Maximum LDH release (OD @490 nm – 620 nm)]. Data indicate average ± SEM from n=3 replicates and representative experiment (n = 2) is shown.

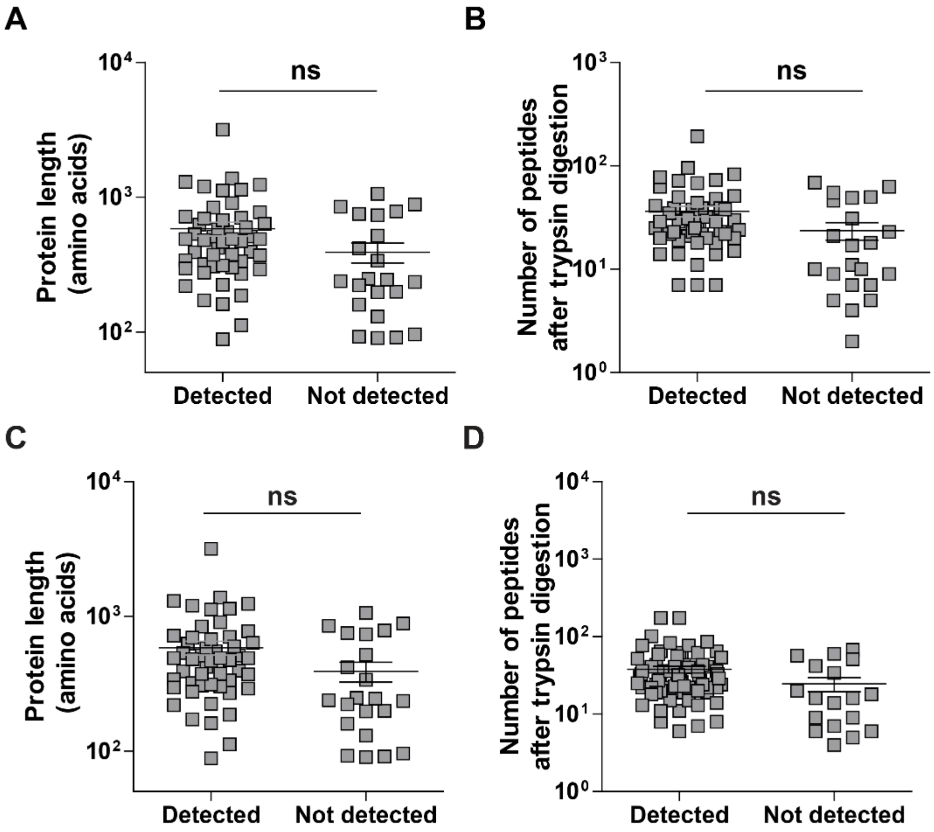

Supplementary Figure 13. Characteristics of virus proteins detected and not detected by mass-spectrometry. Protein length and number of predicted peptides obtained after *in silico* trypsin digestion for HSV-1 proteins (**A-B**) and VZV proteins (**C-D**). ns, not significant; p >0.05 by Unpaired Student's t-test. Grey square: individual HSV-1 proteins. Average ± SD are indicated.

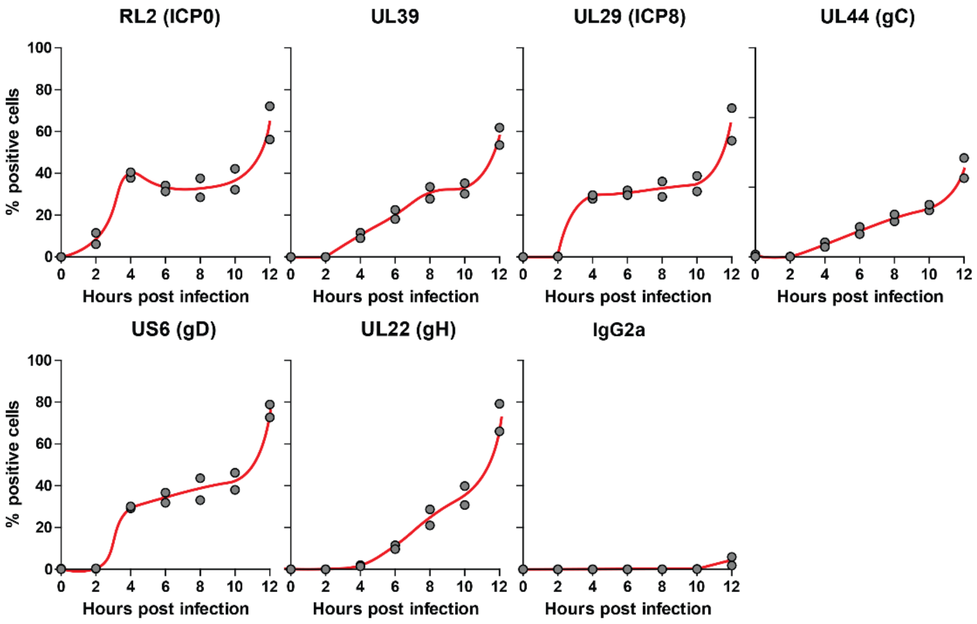

Supplementary Figure 14. Temporal analysis of selected HSV-1 proteins during productive infection of ARPE-19 cells by flow cytometry. HSV-1-infected ARPE-19 cells (F-strain, MOI=1) were analyzed by flow cytometry using antibodies directed to the indicated 6 HSV-1 proteins or IgG2a isotype control. Gates were set based on staining of mock-infected cells and the frequency of cells staining positive for the respective protein is shown. Points indicate individual samples, with n=2 independent experiments per time point. Red line indicates the mean.

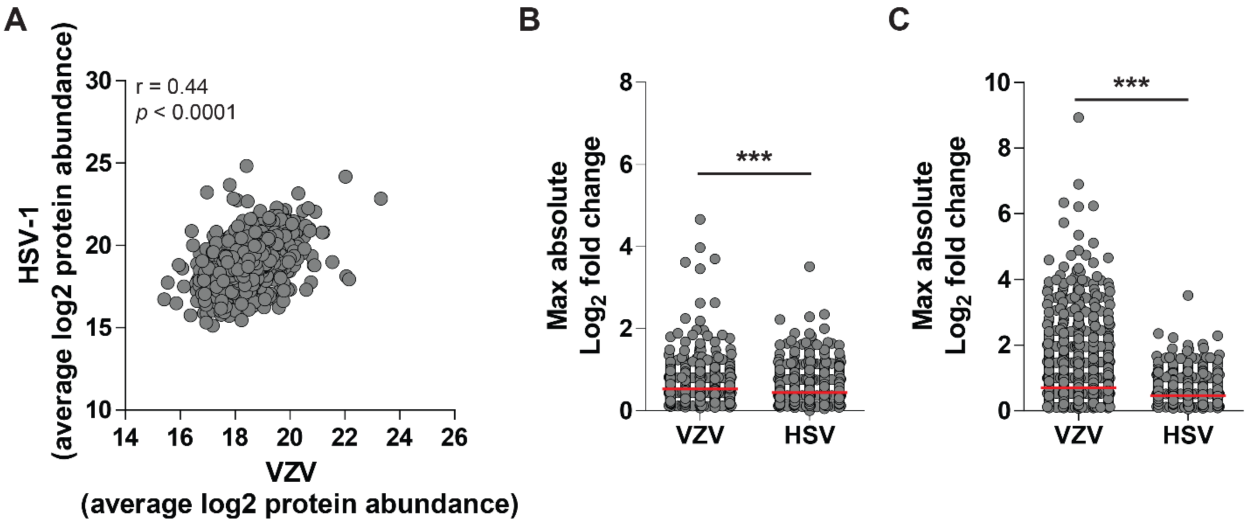

Supplementary Figure 15. Comparison of proteomic HSV and VZV databases. **(A)** Correlation between 1,439 host proteins detected in both HSV and VZV proteomic databases. Spearman r and p-value are indicated. **(B-C)** Comparison of maximum absolute Log2-fold change protein expression between VZV and HSV proteomic databases, including only the 1,439 host proteins detected in both databases **(B)** or all host proteins in each respective database **(C)**. $p < 0.001$ by Mann-Whitney U test. Red line indicates median.
